# Systematic Review and Meta-Analysis of the Diagnostic Accuracy of a Graded Gait and Truncal Instability Rating in Acutely Dizzy and Ataxic Patients

**DOI:** 10.1007/s12311-024-01718-6

**Published:** 2024-07-11

**Authors:** Carlos Martinez, Zheyu Wang, Guillermo Zalazar, Sergio Carmona, Jorge Kattah, Alexander Andrea Tarnutzer

**Affiliations:** 1 Hospital Jose Maria Cullen, Santa Fe, Argentina; 2 Division of Quantitative Sciences, Sidney Kimmel Comprehensive Cancer Center, Johns Hopkins University School of Medicine, Baltimore, MD USA; 3 Department of Biostatistics, Johns Hopkins Bloomberg School of Public Health, Baltimore, MD USA; 4 Hospital de San Luis, Fundación San Lucas Para La Neurociencia, Rosario, Argentina; 5 Fundación San Lucas Para La Neurosciencia, Rosario, Argentina; 6 University of Illinois College of Medicine, Peoria, IL USA; 7 Faculty of Medicine, University of Zurich, Zurich, Switzerland; 8 Neurology, Cantonal Hospital of Baden, Baden, Switzerland

**Keywords:** Truncal instability, Ataxia, Stroke, Vestibular, Vertigo

## Abstract

**Background:**

In patients presenting with acute prolonged vertigo and/or gait imbalance, the HINTS [Head-Impulse, Nystagmus, Test-of-Skew] are very valuable. However, their application may be limited by lack of training and absence of vertigo/nystagmus. Alternatively, a graded gait/truncal-instability (GTI, grade 0–3) rating may be applied.

**Methods:**

We performed a systematic search (MEDLINE/Embase) to identify studies reporting on the diagnostic accuracy of bedside examinations in adults with acute vestibular syndrome. Diagnostic test properties were calculated for findings using a random-effects model. Results were stratified by GTI-rating used.

**Results:**

We identified 6515 articles and included 18 studies (*n* = 1025 patients). Ischemic strokes (*n* = 665) and acute unilateral vestibulopathy (*n* = 306) were most frequent. Grade 2/3 GTI had moderate sensitivity (70.8% [95% confidence-interval (CI) = 59.3–82.3%]) and specificity (82.7 [71.6–93.8%]) for predicting a central cause, whereas grade 3 GTI had a lower sensitivity (44.0% [34.3–53.7%] and higher specificity (99.1% [98.0–100.0%]). In comparison, diagnostic accuracy of HINTS (sensitivity = 96.8% [94.8–98.8%]; specificity = 97.6% [95.3–99.9%]) was higher. When combining central nystagmus-patterns and grade 2/3 GTI, sensitivity was increased to 76.4% [71.3–81.6%] and specificity to 90.3% [84.3–96.3%], however, no random effects model could be used. Sensitivity was higher in studies using the GTI rating (grade 2/3) by Lee (2006) compared to the approach by Moon (2009) (73.8% [69.0–78.0%] vs. 57.4% [49.5–64.9%], *p* = 0.001).

**Conclusions:**

In comparison to HINTS, the diagnostic accuracy of GTI is inferior. When combined with central nystagmus-patterns, diagnostic accuracy could be improved based on preliminary findings. GTI can be readily applied in the ED-setting and also in patients with acute imbalance syndrome.

**Supplementary Information:**

The online version contains supplementary material available at 10.1007/s12311-024-01718-6.

## Introduction

Between 2.1% and 4.4% of all emergency department (ED) consultations are due to a presenting symptom of acute vertigo or dizziness [1–3]. In numbers, this results in approximately 4.4 million visits to US EDs every year, with associated healthcare costs surpassing $10 billion [4]. Whereas the majority of patients will be diagnosed with benign and self-limiting (mostly peripheral) diseases, at least 15% of all ED patients with these symptoms suffer from a dangerous underlying cause [1]. The most common dangerous cause is stroke, accounting for ~ 3–5% of cases [5].

For patients meeting diagnostic criteria for an acute vestibular syndrome (AVS, defined as a clinical syndrome of acute-onset, continuous vertigo, dizziness, or unsteadiness lasting days to weeks, and generally including features suggestive of new, ongoing vestibular system dysfunction (e.g., vomiting, nystagmus, severe postural instability) [6]) a structured approach has been promoted [5]. This includes the use of targeted neuro-otologic bedside examination protocols such as HINTS (Head Impulse test, Nystagmus exam, Test of Skew) [7], HINTS + (which adds a bedside test of hearing) [8], and assessing for gait/truncal instability [9, 10]. While HINTS( +) have an excellent diagnostic accuracy for distinguishing benign, self-limited peripheral causes from dangerous, potentially life-threatening central causes (mostly strokes) when performed by appropriately trained clinicians, being superior than MRI-DWI in the first 24–48 h [11], its application by frontline health-care providers such as ED physicians or neurology residents lacking dedicated training may be not be feasible. In these circumstances, other bedside examination techniques that are more accessible to frontline healthcare providers must be considered instead [10]. This may include both the assessment of (spontaneous) nystagmus patterns by frontline providers and a graded gait and truncal instability (GTI) rating. Previously, we have reported on the diagnostic accuracy of spontaneous nystagmus (SN) patterns in AVS patients [12]. In this systematic review and meta-analysis, we demonstrated a high specificity (97.7% [95% CI = 95.1–100.0%]), but a low sensitivity (19.1% [10.5–27.7%]) for SN patterns predicting a central cause (i.e., isolated torsional SN or (isolated) vertical SN). Thus, the value of an SN analysis in the ED assessment of AVS patients is limited, as only a minority of AVS patients with an underlying central cause will present with a central-type SN pattern. Alternatively, the use of a graded GTI rating has been promoted to distinguish between peripheral and central causes of AVS, with two similar ratings systems being published and applied in several studies [13, 14]. Thereby the patient’s ability to walk and stand unsupported are rated, using a scale from 0 (no gait ataxia or truncal instability) to 3 (severe truncal instability, not being able to stand or sit unassisted). In a recent systematic review and meta-analysis, the diagnostic accuracy of a truncal/gait ataxia assessment in acutely dizzy patients presenting to the ED was reported [15]. In this review, including a total of 1810 patients from 10 studies, a pooled sensitivity of 69.7% (95% CI 43.3%–87.9%) and a pooled specificity of 83.7% (95% CI 52.1%–96.0%) was reported for predicting a central cause. Importantly, only 5 out of 10 studies explicitly defined truncal/gait ataxia and the granularity of GTI assessment amongst studies varied substantially. Whereas some studies provided a graded truncal/gait ataxia rating (grade 0–3), others only distinguished between truncal/gait ataxia being present or absent. Considering these limitations, no conclusion on the diagnostic accuracy of a graded GTI rating in acutely dizzy patients can be drawn from this systematic review. Focusing on high-quality (level of evidence 1–3) studies that also provided a HINTS assessment, we reported a high specificity (99.2% (97.8–100%) and a low sensitivity (35.8% (5.2–66.5%) for a grade 3 GTI rating (severe gait/truncal instability) for predicting a central cause [11]. Noteworthy, in this systematic review, only three studies were included for this assessment. Thus, while the application of a graded GTI assessment in the acutely dizzy patient seems to be a promising alternative approach when the use of oculomotor bedside testing (such as HINTS [7]) is not feasible, this observation is based on a relatively small data sample.

Thus, the primary aim of this systematic review and meta-analysis was to report on the prevalence and diagnostic yield of a graded GTI rating in acutely dizzy patients, using a more inclusive approach in order to increase the number of eligible patients and studies. As recently proposed by Carmona and colleagues [16], we will also assess the diagnostic accuracy of a combined rating, including both the GTI grade and the presence / absence of a central-type (spontaneous or gaze-evoked) nystagmus. Furthermore, we also asked the question, to which extent the grading system used and the vascular territory (in ischemic strokes) involved has an impact on the diagnostic accuracy of the GTI rating. We hypothesized that the presence of grade 2 or grade 3 GTI is predictive for a central cause of AVS, with adding information on the nystagmus pattern to this rating increasing its diagnostic accuracy further.

## Methods

### Data Sources and Searches

We searched MEDLINE and Embase for English-language articles, using the following strategy and components: (1) vertigo/dizziness, (2) diagnostic accuracy of gait/truncal instability as assessed at the bedside, and (3) acute vestibular syndrome (including ischemic stroke, acute peripheral vestibulopathy). We also performed a manual search of reference lists from eligible articles and contacted corresponding authors where necessary. We did not seek to identify research abstracts from meeting proceedings or unpublished studies. We limited our search to articles published since 2002, covering the last 22 years. Our search was updated through June 5th, 2024. This review is reported in accordance with PRISMA guidelines and has been registered in PROSPERO (CRD42022372383). Being a systematic review of the literature and a meta-analysis, no ethical approval was necessary.

### Study Selection and Quality Assessment

Articles were selected by two independent screeners using pre-determined inclusion criteria and a structured process (see supplementary file 1 for details). Our focus was on studies examining the diagnostic accuracy of gait/truncal instability for distinguishing between peripheral and central causes of AVS in patient populations in the ED or on an acute inpatient ward in the acute stage (i.e., within 72 h). Testing of all patients included within 72 h was achieved in seven studies [8, 16–21]. Four studies did not provide any information on the delay from symptom onset to bedside testing [13, 22–24], the remaining seven studies provided mean or median values of delay from symptom-onset to bedside testing only. Thus, a subset of patients may have received delayed (i.e., after more than 72 h) examination only (see Supplementary file 3, Table S3-1 for more details).

We calculated interrater agreement on full-text inclusion using Cohen’s kappa [25]. We assessed the risk of bias and applicability concerns for all studies using QUADAS-2 criteria [26]. The reference standard for “ruling out” stroke in a peripheral vestibular case was delayed (i.e., more than 48 h after symptom onset) magnetic resonance imaging with diffusion-weighted images (MRI-DWI); strokes could be “ruled in” using confirmatory neuroimaging, including computed tomography (CT) in the appropriate clinical context, but an unconfirmed clinical diagnosis was considered high risk of bias.

### Data Extraction, Synthesis and Analysis

We report the diagnostic accuracy of a graded gait and truncal instability (GTI) rating obtained at the bedside. In the literature two different rating scales have been established, both providing a semiquantitative assessment with 4 different grades (GTI 0–3) [13, 14], which are described in detail in Table 1. Furthermore, we also retrieved information on other bedside neuro-otologic assessments including the head-impulse test, alternating cover test (looking for a skew deviation), gaze-holding at eccentric gaze (looking for a horizontal, bidirectional gaze-evoked nystagmus) and spontaneous (horizontal, vertical or torsional) nystagmus at primary gaze. For spontaneous nystagmus, we will distinguish between patterns that do not allow a distinction between peripheral and central causes (such as horizontal or horizontal-torsional patterns) and patterns that are suggestive for a central cause such as (isolated) vertical SN or isolated torsional SN [12]. While presence of (horizontal-torsional) SN is a defining criterion for acute unilateral peripheral vestibulopathy [27], it is observed in only about half of patients with central causes of AVS [12]. Thus, absence of any horizontal SN makes a central cause of the patient’s acute persistent vertigo or dizziness more likely. Overall, central-type nystagmus patterns considered here are (isolated) vertical, torsional-vertical or isolated torsional SN and gaze-evoked nystagmus.

**Table 1:** Graded rating of gait and truncal instability (GTI)

Grade of gait inability	Definition
0	Normal gait, i.e. being able to stand on tandem Romberg with the eyes open for 3 s [13] or no imbalance while walking independently [14]
1	Mild to moderate imbalance but can walk independently [14] or unable to stand on tandem Romberg with the eyes open at least for 3 s [13]
2	Severe imbalance with standing and cannot walk without support [14], or unable to stand on Romberg with eyes open for 3 s [13]
3	Inability to stand upright unassisted [13, 14] or inability to sit upright unassisted [13]

We also evaluated the impact of the lesion location (e.g. in the vascular territory of the posterior inferior cerebellar artery [PICA] vs. the anterior inferior cerebellar artery [AICA]) on the diagnostic accuracy of the different bedside tests evaluated.

We calculated sensitivity, specificity, negative likelihood ratio (LR-) and positive likelihood ratio (LR +) for ED or acute-ward diagnoses for any central condition (including stroke, but not limited to this entity as the clinical presentation of other causes of cAVS is often overlapping (e.g. vestibular migraine)). We present estimated proportions and, where appropriate, 95% confidence intervals (95% CI). Confidence intervals for sensitivities and specificities are calculated based on the Wilson method for binomial counts [28]. For a study with zero cells, a continuity correction of 0.5 is added to all cells of that study. Confidence intervals for positive and negative likelihood ratios are calculated as suggested by Simel and colleagues [29]. A summary measure for each “finding” was calculated using a random effect model using the DerSimonian-Laird estimator [30, 31]. Sensitivity of a study was calculated (and contributed to the random effect model) even when all other measures were not available (missing false-positives or true-negatives). Tests of heterogeneity were conducted based on Cochran’s Q-test. Heterogeneity statistics (Cochran’s Q-test) and comparison of proportions (Fisher’s exact test, [32]) were calculated using R v4.2.1 (Foundation for statistical computing, Vienna, Austria) by a PhD biostatistician. Being a systematic review, no ethical approval was necessary for this study.

### Data Availability

Source data used for meta-analysis will be made available to others upon request to the corresponding author.

## Results

### Characteristics of Studies and Patients

**Fig. 1:**
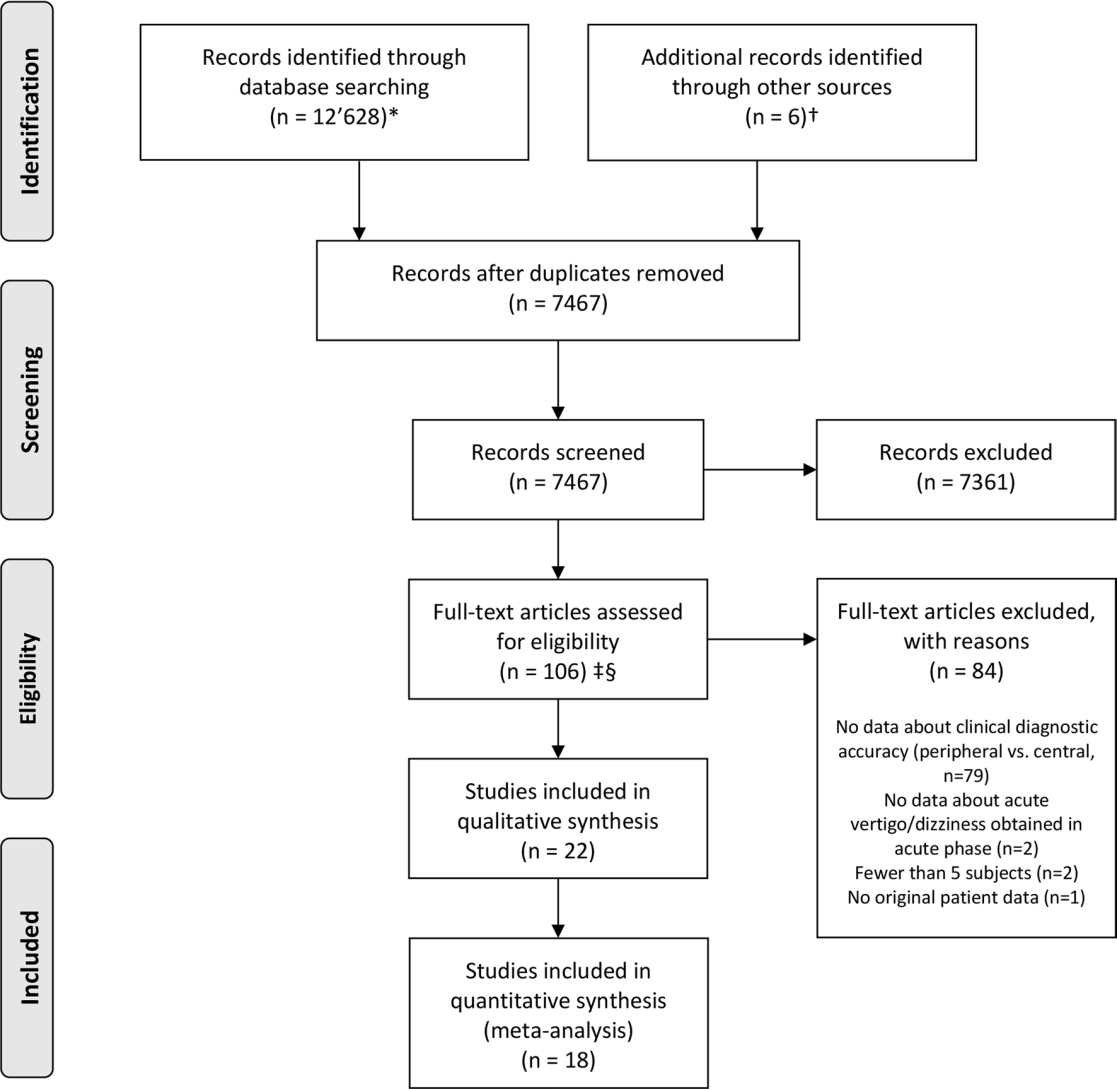
Citation search and selection flow diagram. *MEDLINE was accessed via PubMed; Embase was accessed via embase.com. †Hand search of citation lists from selected studies and investigator files identified 6 additional manuscripts for review. ‡Abstracts coded as “yes” or “maybe” by at least one reviewer were included in full-text review. §After full-text evaluation by two reviewers, any differences were resolved by discussion and adjudication by a third, independent reviewer

Our search identified 7′467 unique citations, of which 7361 (98.7%) were excluded at the abstract level. Our independent raters had high initial agreement on inclusion of full-text manuscripts (kappa value 0.85). After resolving initial disagreements, 22/106 studies were considered eligible (Fig. 1 – PRISMA flow chart), representing 0.3% of the total. Level of evidence varied substantially amongst studies (LOE1 = 1 studies; LOE2 = 1; LOE3 = 3; LOE4 = 15). Among the 1 studies included in the final meta-analysis (one study reporting preliminary data [7] and three studies that did not provide suitable data sets for analysis [33–35] were excluded), the risk of bias and applicability concerns using the QUADAS-2 rating system was judged ‘high’ or ‘unclear’ in one (*n* = 3), two (*n* = 6), three (*n* = 2) or more than three (*n* = 4) of the seven QUADAS-2 bias/applicability categories (see Supplementary file 2, Table S2-1).

Studies included for the quantitative analysis (*n* = 18) reported on 1025 unique patients (665 ischemic strokes; 9 intracranial hemorrhages; 35 other central causes (including vestibular migraine, multiple sclerosis and Wernicke encephalopathy); 306 acute unilateral vestibulopathy; and 10 other peripheral-vestibular causes (including Ramsey-Hunt syndrome and labyrinthine ischemia), full study details are provided in Table 2. From eight studies we retrieved data on mixed populations with both peripheral and central causes, whereas in the other ten studies we obtained data from cAVS patients only. The study design was cross-sectional in all 18 studies and data collection was prospective in the majority (*n* = 12 studies). Bedside testing was performed most frequently by expert neuro-otologists (10 out of 18 studies), but also trained neurologists, neurology-residents with super-vision and general neurologists without sub-specialty were involved (see Table 2 for details). Information how gait / truncal instability was obtained was provided in all 18 manuscripts. A detailed grading (grade 0–3), however, was reported in 14 studies only, with reference to an established grading system in 13 manuscripts (either referring to [14] (*n* = 7) or [13] (*n* = 6). In the remaining four studies [17, 21, 23, 36], only a distinction was made whether patients could stand unassisted or not (i.e. GTI grade 0/1/2 vs. GTI grade 3 according to Lee et al. [14]).

**Table 2:** Key epidemiologic aspects

	**cAVS (n subjects)**	**pAVS (n subjects)**
*Gender*		
Women	244	141
Men	401	144
Not reported	64	31
Total	709	316
*Diagnoses*		
Ischemic stroke	665	NA
PICA territory	287	NA
AICA territory	69	NA
SCA territory	19	NA
Brainstem	165	NA
Other^a^	11	NA
Not specified	114	NA
Hemorrhagic stroke	9	NA
Multiple sclerosis	11	NA
Vestibular migraine	9	NA
Other^b^	15	NA
Acute vestibular neuropathy	NA	306
Ramsey Hunt Syndrome	NA	7
Labyrinthine ischemia	NA	2
Labyrinthitis	NA	1
	**n [studies, subjects]**	
*Study design*		
Prospective cross-sectional	[12, 724]	
Retrospective cross-sectional	[6, 301]	
*Study target group*		
cAVS population	[10, 341]	
Mixed cAVS and pAVS population	[8, 684]	
*Assessment of GTI – scale used*		
Grade 0–3 according to Lee et al. 2006 [8, 9, 14, 16, 18, 20, 24]^c^	[7, 651]	
Grade 0–3 according to Moon et al. 2009 [13, 19, 22, 37–39]	[6, 155]	
Simplified grading (Grade 0/1/2 vs. Grade 3) [17, 21, 23, 36]	[4, 211]	
Other (own) [40]	[1, 7]	
*Assessment of GTI—examiner*		
Experienced neuro-otologists	[10, 643]	
Neurologists, no specific training in neuro-otology indicated	[6, 244]	
Trained neurology residents with supervision by experienced neuro-otologists	[1, 114]	
Neurologists with 4 h of training in neuro-otology	[1, 24]	
*Bedside vestibular testing performed*		
Horizontal head impulse test [8, 9, 13, 14, 17–19, 22–24, 37, 38]	[12, 652]	
Direction-changing nystagmus on lateral gaze [8, 9, 14, 17–19, 22–24, 37, 38, 40]	[12, 651]	
Alternating cover test [8, 9, 17–19, 22–24, 36, 38]	[10, 738]	
HINTS battery [8, 9, 17–19, 22, 24, 38]	[8, 574]	
HINTS-plus battery [8, 9, 17, 18, 22]	[5, 390]	
Vertical or torsional SN [8, 9, 13, 14, 16, 17, 19, 20, 24, 37, 38, 40]	[12, 711]	
Babinski asynergy sign [9, 20]	[2, 60]	
Incubitus truncal ataxia [20]	[1, 11]	

*Abbreviations*: *AICA* Anterior inferior cerebellar artery, *AVS* Acute vestibular syndrome, *BPPV* Benign paroxysmal positional vertigo, *cAVS* Central AVS, *ED* Emergency department, *ENT* Ear nose throat, *GTI* Gait and truncal instability, *HINTS* Head Impulse test, Nystagmus examination, Test of Skew, *HINTS plus* HINTS plus new unilateral hearing loss, *pAVS* peripheral AVS, *PICA* Posterior inferior cerebellar artery, *SCA* Superior cerebellar artery, *SN* Spontaneous nystagmus, *SSNHL* Sensorineural hearing loss

^a^Other ischemic stroke locations included strokes within multiple territories (*n* = 6), thalamic strokes (*n* = 3) and strokes in the territory of the middle cerebral artery (MCA) (*n* = 2)

^b^Other central causes included Wernicke encephalopathy (*n* = 4), intoxication with antiepileptic drugs (*n* = 3), undefined ataxia under evaluation (*n* = 3), cerebellar tumors (*n* = 2), paraneoplastic (*n* = 2) and brainstem encephalitis (*n* = 1)

^c^From one study, truncal ataxia ratings were not previously published and were provided via personal communication with the corresponding author [8]

In addition to the reported GTI ratings, some studies also provided assessments of other vestibulospinal signs (Babinski asynergy sign, incubitus truncal ataxia) and oculomotor bedside examinations, including the ability to hold gaze in an eccentric position (i.e., reporting on gaze-evoked nystagmus, GEN), vertical divergence on the alternating cover test (i.e., skew deviation, SD), the integrity of the angular vestibulo-ocular reflex by bedside horizontal head-impulse testing (hHIT), and primary gaze, spontaneous nystagmus (see Table 2 for details). The Babinski asynergy sign is assessed by evaluating the patient’s ability to sit up from supine position without using the arms. This sign was considered positive by Babinski in 1913 when the patient was unable to do this. Inability to sit unassisted was referred to as positive “incubitus truncal ataxia” sign in one study [20].

### Diagnostic Accuracy of the Gait and Truncal Instability Rating and Comparison to Other Bedside Tests

**Fig. 2:**
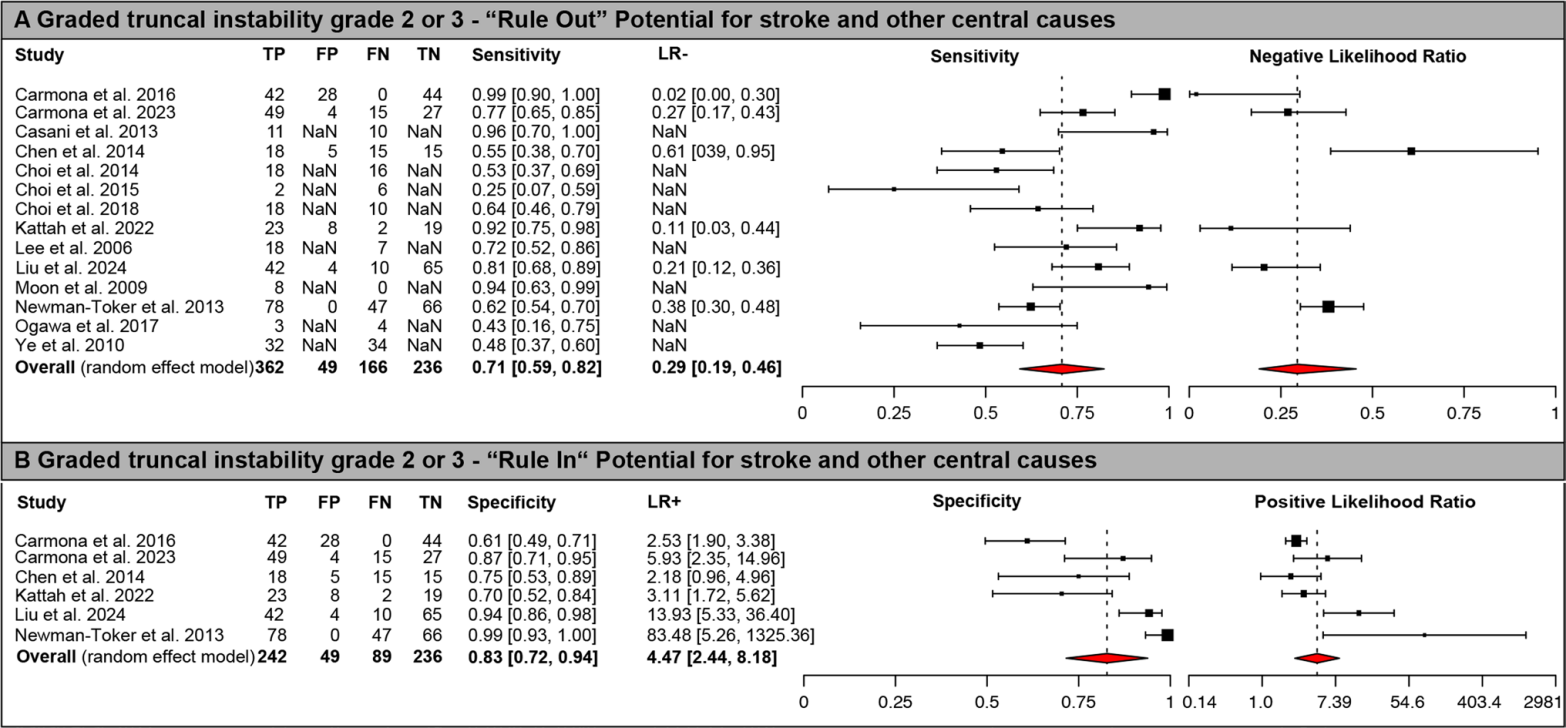
Forest plots of diagnostic test properties. Forest plots of diagnostic test properties for a graded gait/truncal instability (GTI) ratings comparing Grade 0/1 vs. 2/3. Panel A – sensitivity and negative likelihood ratio (LR-) including 95% confidence intervals (CI); Panel B – specificity and positive likelihood ratio (LR +) including 95% CI. Single study results and aggregated values for all test properties (including 95% CI) are provided for all studies included (*n* = 17). Summary measures were calculated using a random effects model using the DerSimonian-Laird estimator [30, 31]. Heterogeneity amongst all studies was significant both for ruling out (i.e., sensitivity, *p* < 0.001) and for ruling in (i.e., specificity, *p* < 0.001) central (mostly vertebrobasilar ischemic stroke) causes using Cochran’s Q. Note that the axis for positive likelihood ratio uses an exponential scale

The diagnostic accuracy of different cut-offs of graded gait and truncal instability for predicting a central cause of AVS were compared. When considering only severe (grade 3) GTI as an indicator of a central cause of AVS, the sensitivity was low (44.0% [95% confidence-interval (CI) = 34.3–53.7%]), whereas the specificity was very high (99.1% [98.0–100.0%]). Being more inclusive, i.e., taking into account either grade 2 or grade 3 GTI, the sensitivity for predicting a central cause of the patient’s AVS increased to a moderate value (70.8% [59.3–82.3%]) and the specificity decreased (82.7% [71.6–93.8%]), as shown in detail in the forest plots in Fig. 2. When considering both grade 1, 2 and 3 GTI as indicative of a central cause, sensitivity further increased, whereas specificity dropped again (see Table 3 for details).

**Table 3:** diagnostic accuracy of selected bedside diagnostic tests in patients with acute vestibular syndrome

Bedside diagnostic tests	Sensitivity % (95% CI)	LR- (95% CI)	Specificity % (95% CI)	LR + (95% CI)
**Truncal instability (grade 1/2/3)**^a^ [8, 9, 13, 14, 16, 18–20, 22, 36–40]	91.8 (88.0–95.6)	0.25 (0.19–0.33)	25.7 (0.0–73.9)	1.07 (0.98–1.16)
**Truncal instability (grade 2/3)**^a^ [8, 9, 13, 14, 16, 18–20, 22, 24, 37–40]	70.8 (59.3–82.3)	0.30 (0.19–0.46)	82.7 (71.6–93.8)	4.47 (2.44–8.18)
**Truncal instability (grade 3)**^b^ [8, 9, 13, 14, 16–24, 36–40]	44.0 (34.3–53.7)	0.55 (0.43–0.71)	99.1 (98.0–100.0)	29.61 (10.78–81.32)
**Truncal instability and/or central-type nystagmus (grade 1/2/3)**^a^,^c^**,**^d^ [8, 13, 14, 19, 20, 37, 38, 40]	88.2 (84.3–92.1)	0.17 (0.12–0.24)	69.9 (60.6–79.2)	2.93 (2.14–4.01)
**Truncal instability and/or central-type nystagmus (grade 2/3)**^a^,^c^**,**^d^ [8, 13, 14, 19, 20, 37, 38, 40]	77.2 (72.1–82.3)	0.25 (0.20–0.32)	90.3 (84.3–96.3)	7.98 (4.27–14.89)
**Truncal instability and/or central-type nystagmus (grade 3)**^b^,^c^**,**^d^ [8, 13, 14, 17, 19, 20, 37, 38, 40]	61.9 (56.1–67.7)	0.41 (0.35–0.48)	93.5 (88.8–98.1)	9.46 (4.60–19.48)
**HINTS plus** [8, 9, 17, 18, 22]	98.9 (97.5–100.0)	0.03 (0.01–0.11)	95.1 **||** (90.0–100.0 **||**)	12.50 (3.05–51.20)
**HINTS** [8, 9, 17–19, 22, 38]	96.8 (94.8–98.8)	0.07 (0.03–0.14)	97.6 (95.3–99.9)	17.83 (7.63–41.64)
**Normal hHIT** [8, 9, 13, 14, 17–19, 22–24, 37, 38]	91.4 (87.4–95.4)	0.17 (0.13–0.23)	99.1 (98.0–100.0)	31.82 (9.91–102.20)
**Direction-changing nystagmus**^e^ [8, 9, 14, 17–19, 22–24, 37, 38, 40]	26.5 (17.2–35.8)	0.71 (0.62–0.83)	99.2 (98.1–100.0)	22.05 (7.04–69.08)
**Skew deviation** [8, 9, 17–19, 22–24, 36, 38]	20.2 (10.1–30.2)	0.82 (0.72–0.93)	98.0 (96.2–99.8)	6.86 (3.53–13.32)
**No spontaneous horizontal nystagmus** [8, 9, 13, 14, 16–20, 22, 23, 37–40]	46.7 (35.1–58.3)	0.55 (0.45–0.68)	98.8 (97.3–100.0)	14.52 (3.83–55.12)
**Babinski asynergy sign**^d^ [9, 20]	81.7 (71.9–91.5)	0.19 (0.15–0.24)	97.8 (94.8–100.0)	37.16 (26.82–51.48)

*Abbreviations*: *AICA* Anterior inferior cerebellar artery, *APV* Acute peripheral vestibulopathy, *cAVS* Central acute vestibular syndrome, *HINTS* Head-impulse, nystagmus, test of skew, *HINTS plus* Head-impulse, nystagmus, test of skew, new hearing loss, *hHIT* Horizontal head-impulse test, *LR* + Positive likelihood ratio, *LR-* Negative likelihood ratio, *pAVS* peripheral acute vestibular syndrome, *PICA* Posterior inferior cerebellar artery, *SN* Spontaneous nystagmus

^*^Only a subset of studies provided all items to calculate the HINTS “plus”. Adding a fourth item (Boolean “or”) to a decision rule (compared to the three-item HINTS [without hearing]) can only decrease the specificity, thus specificity values for HINTS “plus” will be equivalent or slightly lower

^a^Different ratings for ataxia grade 1 and 2 were used in these studies. While some studies adhered to the GTI definitions as proposed by Moon and colleagues [13], who defined grade 1 as "sway on Romberg" and grade 2 as "able to stand but no tandem gait" [13, 19, 22, 37–39], other studies followed the GTI definitions as proposed by Lee and colleagues [14], who defined grade 1 as "mild to moderate imbalance with walking independently" and grade 2 as "severe imbalance with standing, but cannot walk without support" [8, 9, 14, 16, 18, 20, 24]

^b^There were minor differences in the definition of grade 3 truncal ataxia amongst different studies. While Lee and colleagues defined grade 3 GTI as "falling at upright posture" [14], Moon and colleagues referred to grade 3 GTI as “unable to stand or sit without support” [13]

^c^Central-type nystagmus was defined as purely vertical, purely torsional, combined vertical-torsional (including seesaw nystagmus), horizontal-vertical spontaneous nystagmus, periodic alternating nystagmus (PAN), or gaze-evoked nystagmus

^d^For these combined bedside examination approaches, too few studies provided all the numbers required to calculate reliable results using the random effect model (using the DerSimonian-Laird estimator). Therefore, these results are not shown here and non-model based (preliminary) calculations for sensitivity, specificity, LR + and LR- are shown here instead (using excel instead). Note that the results of these calculations do not have the same rigor and thus cannot be compared directly with the other diagnostic accuracy measures

^e^Only studies that explicitly reported on directional changes of spontaneous nystagmus in left vs. right gaze were included, whereas studies reporting on the mere “presence vs. absence” of gaze-evoked nystagmus without providing the laterality or direction of the nystagmus were discarded. This is because unilateral gaze-evoked nystagmus (or bilateral gaze-evoked nystagmus, beating in the same direction) would not meet the criteria for this clinical finding

In comparison to the GTI ratings, sensitivity for detecting a central cause of AVS was highest for a normal hHIT (91.4% [95% CI 87.4–95.4]), but was considerably lower for the other tests including the test of skew (20.2% [10.1–30.2]), direction-changing nystagmus on lateral-gaze test (26.5% [17.2–35.8]) and absence of any spontaneous horizontal nystagmus (46.7% [35.1–58.3]). Specificity for detecting a dangerous central cause of AVS was high for all these bedside tests (see Table 3 for details).

**Fig. 3:**
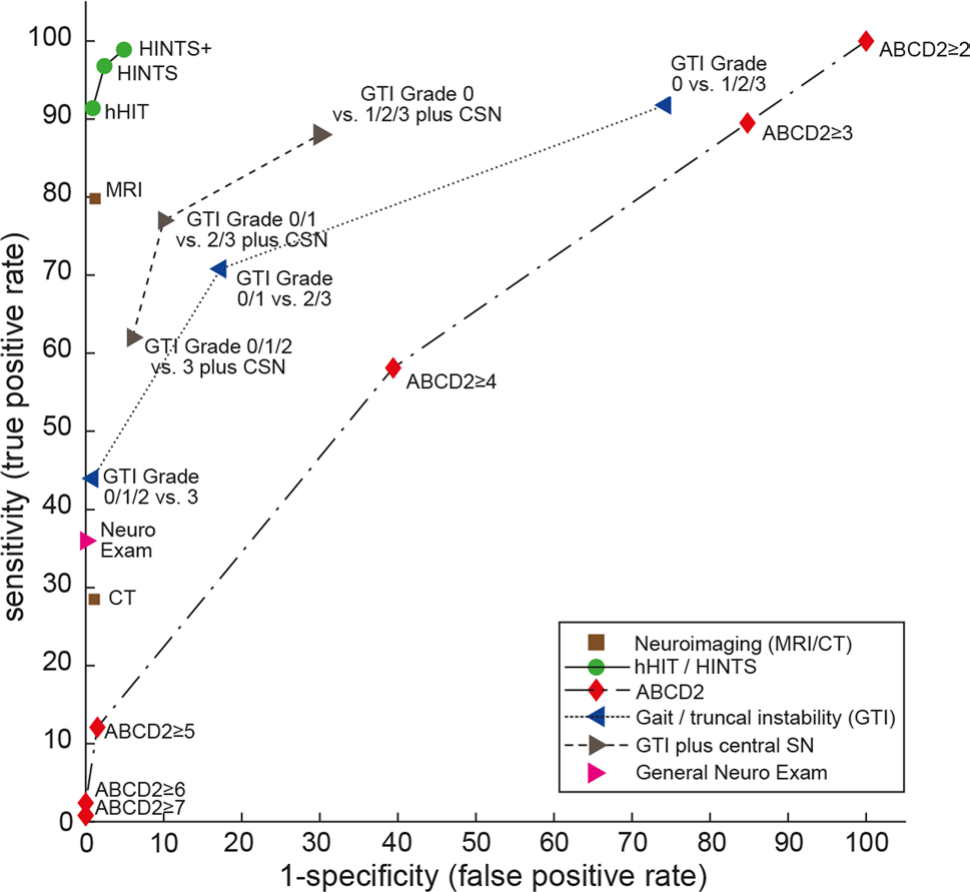
Summary receiver operating characteristic curve analysis for various bedside tests compared to brain imaging and clinical scores. Summary receiver operating characteristic (SROC) curve analysis for the graded gait/truncal instability (GTI) ratings (with or without additional central-type spontaneous/gaze-evoked nystagmus compared to the “HINTS (Head Impulse, Nystagmus, Test of Skew) family”, neuroimaging (computed tomography [CT] or magnetic resonance imaging with diffusion-weighted sequences [MRI-DWI], values used as published by Shah and colleagues [41]), general neurologic exam and vascular risk stratification by ABCD2 (age, blood pressure, clinical features, duration of symptoms, diabetes) score (data from a single study [8]) for detecting stroke in patients presenting the acute vestibular syndrome (modified after [42]). SROC curves are shown for six different diagnostic approaches to diagnosing stroke in the acute vestibular syndrome. A perfect test or decision rule has threshold cutoffs in the upper left corner (100% sensitivity, 100% specificity) and an area under the curve (AUC) of 1.0. Note that the gait/truncal instability ratings outperform the ABCD2 score and the general neurologic exam but are inferior compared to the HINTS family of eye movement tests. This is also true when adding central-type spontaneous nystagmus to the GTI rating. Both HINTS and HINTS plus (HINTS plus new hearing loss detected by finger rubbing or similar) demonstrate a higher diagnostic accuracy for ruling out stroke than MRI including DWI

For the HINTS bedside examination testing battery, the diagnostic accuracy was substantially higher than for any GTI rating (see Fig. 3 and Table 3). When adding a fourth sign (new-onset unilateral hearing loss) to the HINTS (i.e., HINTS “plus”), the sensitivity increased slightly to 98.9% (97.5–100.0%), whereas specificity was somewhat lower.

Overall results plotting sensitivity and specificity for different bedside tests and providing neuroimaging accuracy point estimates for comparison and context are shown in a summary receiver operating characteristic (SROC) curve (Fig. 3). Individual test details are found in Supplementary file 3, Table S3-2. Heterogeneity for sensitivity and specificity (based on Cochran’s Q-Test of heterogeneity) was significant in the majority of bedside tests evaluated (see Supplementary file 3, Table S3-2 for details).

### Combining the GTI Rating with the Presence / Absence of a Central-Type Spontaneous Nystagmus

Data on both GTI grade (0–3) and central nystagmus patterns were available in 8 studies [8, 13, 14, 19, 20, 37, 38, 40] (with an additional study reporting on GTI 0/1/2 vs. 3 only [17]). When calculating the accuracy measures using the random effect model with the DerSimonian-Laird estimator, however, there were too few studies providing a sufficient number of values required for the calculation and the difference among them. Thus, calculated results were not reliable and are not shown here. When calculating sensitivity and specificity without a model-based approach instead, i.e. defining sensitivity as the number of true positives over the sum of all true positives and false negatives and specificity as the number of true negatives over the sum of all true negatives and false positives, sensitivity and specificity values increased compared to when using the GTI rating only. This was true both when requiring GTI grade 3 (and/or central type of SN) as predictor for central, but also when requiring GTI grade 2 or 3 (and/or central type of SN) or GTI grad 1, 2 or 3 (and/or central type of SN) as indicator of a central cause of the patient’s AVS (see Fig. 2 and Table 3).

### Diagnostic Accuracy in AVS Using the GTI Rating by Stroke Location

For the HINTS it has been shown that the diagnostic accuracy significantly depends on the stroke location. Whereas for PICA strokes sensitivity of the HINTS composite examination is very high to detect central causes, it is substantially lower for AICA strokes [11]. We asked whether a similar pattern could be observed for the GTI rating. A detailed stroke location (distinguishing strokes in different vascular territories) was provided in 8 studies (see details in Supplementary file 3 Table S3-2). Sensitivity for detecting stroke when using GTI grade 2/3 was not significantly different for AICA and PICA stroke subtypes (77.4% [63.1–91.6%] vs. 83.5% [73.8–93.2%]), with 95% confidence intervals overlapping. The same was true for GTI grade 1/2/3 and GTI grade 3 only*.*

### Diagnostic Accuracy of Other Bedside Examinations Focusing on Truncal *Ataxia*

A positive Babinski asynergia sign was found in 38/42 cAVS patients (AICA and PICA strokes) in one study [9] and in 11/18 cAVS patients (AICA and PICA strokes) in another study [20], whereas in patients with pAVS a positive Babinski asynergia sign was rarely found (0/72 in one study [9] and 2/19 in the other study [20]). Calculating diagnostic accuracy of the Babinski asynergia sign, sensitivity reached 81.7% [71.9–91.5%], specificity was 97.8% [94.8–100.0%]. However, the number of studies was too small to include this parameter into the random effects model.

A specific comparison between the inability to sit unassisted and the inability to stand unassisted, referred to as the “incubitus truncal ataxia” sign in those patients unable to sit unassisted, was made in a single study only in a total of 11 patients with either AICA or PICA stroke [20]. All 6 strokes (AICA = 4; PICA = 2) that were unable to stand unassisted (i.e. reflecting grade 3 GTI according to Lee et al. [14]) also presented with incubitus truncal ataxia (i.e. inability to sit unassisted). Whereas all AICA strokes studied (*n* = 4) had positive incubitus truncal ataxia, this was the case only in 2/7 PICA strokes.

### The Impact of Different Rating Systems of GTI on Diagnostic Accuracy

We asked whether the different GTI rating systems in use had an impact on the diagnostic accuracy in AVS patients. Overall, the GTI rating system according to Lee et al. [14] was applied in seven studies and the GTI rating system as proposed by Moon et al. [13] was used in six studies. Specifically, 651 patients (cAVS = 366; pAVS = 285) that were graded according to Lee et al. [14] and 155 patients (all cAVS) that were graded according to Moon et al. [13]. Sensitivity was significantly higher in studies using the GTI rating (grade 2/3 vs. grade 0/1) as proposed by Lee and colleagues compared to the approach suggested by Moon and colleagues (73.8% [69.0–78.0%] vs. 57.4% [49.5–64.9%], *p* < 0.001). No comparison of specificity could be made as the GTI rating by Moon et al. was applied in studies only that reported on cAVS patients.

## Discussion

In this systematic review and meta-analysis, we addressed the diagnostic accuracy of a graded gait and truncal instability (GTI) rating in patients with acute vestibular syndrome (AVS) of either peripheral (pAVS) or central (cAVS) origin. The GTI grading can be considered an alternative to the HINTS bedside exam in those patients that either do not present with vertigo or nystagmus (which is mandatory when applying the HINTS) or when the examiner is not trained to perform and interpret the HINTS. In our meta-analysis, the sensitivity and specificity of the GTI grading varied substantially depending on the selected cut-off of the GTI grade to consider it of central origin. Overall, when requiring at least moderate (grade 2) or severe (grade 3) GTI, sensitivity (70.8% [95% confidence-interval (CI) = 59.3–82.3%]) and specificity (82.7% [71.6–93.8%] for predicting a central lesion were intermediate only and clearly below the diagnostic accuracy of HINTS (sensitivity = 96.8% [94.8–98.8%]; specificity = 97.6% [95.3–99.9%]). The location of the ischemic stroke (AICA vs. PICA stroke) had no impact on the diagnostic accuracy on the GTI, whereas using the GTI definitions as proposed by Lee and colleagues [14] compared to those definitions introduced by Moon et al. [13] resulted in a significantly higher sensitivity.

### The Value of a Graded GTI Rating in AVS

In the hands of neuro-otologists and trained emergency department physicians, HINTS and HINTS plus have been shown to have excellent diagnostic accuracy [11]. Physicians that have received little (i.e., less than six hours) or no training at all often struggle in applying the HINTS( +) properly, either using them in the wrong patients, performing the test improperly, or interpreting the results incorrectly [43, 44]. Thus, until training of this group is successfully implemented at a larger scale, other accurate tests that do not rely on subtle oculomotor findings might help [10]. The gait assessment is an established part of the basic standard neurologic exam for a dizzy patient presenting to the ED. With an intermediate diagnostic accuracy when selecting a cut-off of GTI grade 2 as indicative for a central cause, the graded GTI rating is superior to a general neurologic exam or CT-based imaging (see Fig. 2), but about 3 out of 10 cAVS cases are missed. When requiring grade 3 GTI for a central origin, the sensitivity was low (44.0% [34.3–53.7%]), whereas the specificity was very high (99.1% [98.0–100.0%]). Thus, presence of a grade 3 GTI rating is highly suggestive for a central cause, but its absence does not exclude a central origin of the patient’s AVS. Likewise, considering a grade 1, 2 or 3 GTI rating as central will increase the sensitivity of the test, but at the same time will result in many false positive cases (with specificity dropping to 25.7% [0.0–73.9%]). Overall, it is recommended to consider a grade 2 or grade 3 GTI as indicative of a central origin to achieve best diagnostic accuracy.

However, as such the GTI rating is not sufficiently sensitive to identify all central causes and therefore should be combined with other bedside examinations. The presence of a central-type nystagmus (i.e., a purely vertical or torsional, a vertical-torsional or a vertical-horizontal nystagmus at primary gaze) has been shown to have excellent specificity (97.7% [ 95.1–100.0%]), but low sensitivity (19.1% [10.5–27.7%]) in a recent meta-analysis [12]. When combining central nystagmus patterns (either at primary gaze or at eccentric gaze) and grade 2/3 GTI, sensitivity for detecting lesions of central origin was increased to 77.2% [72.1–82.3%] and specificity to 90.3% [84.3–96.3%], however, no random effects model could be used. Therefore, such a combined approach can be considered promising, but results at this time are preliminary only and additional prospective studies looking into the diagnostic accuracy of this approach are needed. Furthermore, it has been recently shown by one of the authors that concordant presence of ocular lateral deviation and severe truncal ataxia (grade 3) is highly suggestive of an ipsilesional medullary syndrome [45]. Thus, further combining different clinical signs needs to be assessed with more detail in future studies.

### Optimizing the Graded Gait and Truncal Instability Rating

We identified two distinct sets of definitions for rating gait and truncal instability. While both grading systems [13, 14] were used in six studies included in our meta-analysis each, a direct comparison of the diagnostic accuracy of both GTI grading systems is only partially possible. Whereas the grading system by Lee and colleagues demonstrated a significantly higher sensitivity than the grading system proposed by Moon and colleagues, no comparison of specificity is possible since no pAVS cases were reported by those studies that implemented the GTI grading system according to Moon and colleagues. Furthermore, a head-to-head comparison of both grading systems in the same patient population is lacking, thus differences in the sensitivity could be related to superior applicability of one grading system, but also to distinct patient populations examined or a varying extent of familiarity with the two grading systems by the examiners involved. With regards to applicability of the GTI grading system in the ED, stopping time – as required for the grading system by Moon and colleagues – could limit its use also. This might be a potential disadvantage for this GTI grading system. While clearly different conditions were assessed for grade 1 and grade 2 GTI, for grade 3 GTI discrepancies between the two rating systems were minor only. Whereas all studies adhering to the rating system proposed by Moon and colleagues considered grade 3 GTI as inability to either sit or stand without support, Lee and colleagues defined grade 3 GTI as inability to stand upright unaided. Potentially, focusing on the inability to sit unaided could lower the sensitivity of the test, as this might still be achieved by the patient while standing may not possible any more. A specific distinction between inability to stand upright unaided (GTI grade 3 according to Lee and colleagues [14]) and an inability to sit upright unaided (so-called incubitus ataxia), however, was provided in a single study only [20]. Kattah and colleagues found that “the presence of standing grade 3 ataxia correlated with Incubitus ataxia and occurred in central vascular lesions” [20]. Possibly, in the recovery phase after a (central) AVS, ability to sit unaided could be achieved earlier than the ability to stand unaided. Thus, while in the hyperacute phase both definitions could be equally sensitive, requiring inability to sit unaided for a grade 3 GTI rating may lower the sensitivity in those cases assessed with more delay. Noteworthy, from the limited data available, no conclusions can be drawn whether grade 3 GTI should be defined as inability to sit unaided or as inability to stand unaided and further studies are needed.

### Limitations

This systematic review and meta-analysis has several limitations that need to be further discussed. First, we observed substantial heterogeneity amongst studies reporting on the GTI rating, which may be related to discrepancies in the GTI rating or to the patient cohorts studied. Second, our patient cohort was enriched with cAVS cases (representing 69.2% of all included cases), whereas overall an estimated fraction of 25% of cAVS cases amongst all AVS cases is assumed [46]. This was due to the fact that we included both studies reporting on unselected AVS cohorts and case series with AVS linked to central (ischemic causes), while no study with pAVS cases only reporting on GTI was identified. Third, timing of GTI testing likely varied amongst studies (personal communication with several corresponding authors), but no further details were provided in the studies included. Whereas some studies applied GTI testing early in the clinical examination, others performed testing only after having the patient to rest for at least 5-10 min. Furthermore, delay of testing after symptom onset was reported in two studies only, with both having testing completed within the first 24 h [8, 9], whereas in all other studies this information was not provided. Thus, differences in the delay of testing and the timing of testing during the neurological exam may have affected the rating in both pAVS and cAVS cases as well. Taking into account the recommendations of the classification committee of the Barany Society on vascular vertigo [47], serial evaluation may be needed, especially if initial imaging is negative of symptom duration is less than 24 h. For most publications included here, there is no information on whether this was done or not. Fourth, severe nausea and/or motion intolerance may prevent adequate testing for grades 1, 2 and 3 GTI in the ED [48]. However, assessing the ability to sit up in the stretcher without holding on to the guard rails can be considered a proxy for the GTI assessment, allowing detection of those patients with severe (i.e., grade 3) GTI who are very likely to have a central cause of their AVS also.

## Conclusions

Selective use of a graded gait and truncal instability (GTI) rating in patients presenting with an acute vestibular syndrome has an intermediate diagnostic accuracy only and thus should be combined with other bedside examination techniques. While ideally GTI should be used in addition to the assessment of the HINTS [24], the latter may not be feasible (due to lack of expertise and training of the physician in charge) or not justified (due to lacking vertigo and nystagmus). Thus, alternative bedside techniques are urgently needed and in this setting the use of the GTI grading approach in combination with an assessment of spontaneous and gaze-evoked nystagmus (looking for central nystagmus patterns) is seems promising, with moderate-to-high sensitivity (77.2% [72.1–82.3%]) and specificity (90.3% [84.3–96.3%]), however, needs further evaluation. Preferentially the GTI grading system by Lee and colleagues should be used as it has been applied to a broader range of AVS patients (including also patients with pAVS) and as it was shown to be significantly more sensitive than the GTI grading system by Moon and colleagues. Unlike the HINTS, the GTI grading system was equally accurate in detecting AICA and PICA strokes. In conclusion, a graded GTI rating should be obtained in all AVS patients and should be combined with other bedside (oculomotor) examinations. In patients presenting with acute truncal ataxia without (spontaneous or gaze-evoked) nystagmus, HINTS may not be applicable. Considering the graded GTI rating instead may therefore provide useful, as recently demonstrated by Carmona and colleagues [16].

## Supplementary Information

Additional file 1.

Additional file 2.

Additional file 3.

## Data Availability

The data that support the findings of this study are available from the corresponding author upon reasonable request.
