# Correction: Systematic Review and Meta-Analysis of the Diagnostic Accuracy of a Graded Gait and Truncal Instability Rating in Acutely Dizzy and Ataxic Patients

**DOI:** 10.1007/s12311-024-01724-8

**Published:** 2024-07-27

**Authors:** Carlos Martinez, Zheyu Wang, Guillermo Zalazar, Sergio Carmona, Jorge Kattah, Alexander Andrea Tarnutzer

**Affiliations:** 1 HospitalJoseMariaCullen, SantaFe, Argentina; 2 Division of Quantitative Sciences, Sidney Kimmel Comprehensive Cancer Center, Johns Hopkins University School of Medicine, Baltimore, MD USA; 3 Department of Biostatistics, Johns Hopkins Bloomberg School of Public Health, Baltimore, MD USA; 4 Hospital de San Luis, Fundación San Lucas Para La Neurociencia, Rosario, Argentina; 5 Fundación San Lucas Para La Neurosciencia, Rosario, Argentina; 6 University of Illinois College of Medicine, Peoria, IL USA; 7 Faculty of Medicine, University of Zurich, Zurich, Switzerland; 8 Neurology, Cantonal Hospital of Baden, Baden, Switzerland

The Cerebellum

10.1007/s12311-024-01718-6

The original version of this article unfortunately contained error in Table 1.

In Table 1 of this paper, the word “tandem” should be removed in “Severe imbalance with standing and cannot walk without support [14], or unable to stand on tandem Romberg with eyes open for 3 s [13]”.

The original article has been corrected.

