# Supplementary material for: Systematic Review and Meta-Analysis of the Diagnostic Accuracy of a Graded Gait and Truncal Instability Rating in Acutely Dizzy and Ataxic Patients
Source: Cerebellum. 2024 Jul 11;23(6):2244–56. doi: 10.1007/s12311-024-01718-6 (PMC11585515; doi:10.1007/s12311-024-01718-6)
Supplement: Supplementary file 1 — Additional file 1. [file 12311_2024_1718_MOESM1_ESM.docx]

## **Supplementary file 1- electronic search strategy, coding-scheme for the systematic review and data analysis**

**The search strategy was designed by a clinical investigator with relevant domain expertise in neurology and in systematic reviews (AAT).**

**We searched MEDLINE and Embase for English-language articles, using the following strategies with the following components: (1) vertigo/dizziness or ataxia, (2) diagnostic accuracy of bedside examination techniques, and (3) acute vestibular syndrome** **(ischemic stroke, acute peripheral vestibulopathy). We also performed a manual search of reference lists from eligible articles and contacted corresponding authors where necessary. We did not seek to identify research abstracts from meeting proceedings or unpublished studies.**

AGGREGATED/COMPOSITE VERSION (June 5th, 2024) [PubMed 6469 abstracts; EMBASE 6159]

((dizz*[tiab] OR vertigo[tiab] OR vestibular[tiab] OR atax*[tiab] NOT “case report”[tiab]) AND (prodrom*[tiab] OR diagnos*[tiab] OR manifestation*[tiab] OR clinical feature*[tiab] OR symptom*[tiab] OR “physical examination”[mh] OR physical exam*[tiab] OR professional competence[mh] OR “sensitivity and specificity”[tiab] OR “sensitivity and specificity”[mh] OR “reproducibility of results”[mh] OR “observer variation”[mh] OR “diagnostic tests, routine”[mh] OR “decision support techniques”[mh] OR “bayes theorem”[mh] OR dizziness/physiopathology[mh] OR vertigo/physiopathology[mh] OR dizziness/diagnosis[mh] OR vertigo/diagnosis[mh]) AND (acute peripheral vestibulopathy[tiab] OR labyrin*[tiab] OR vestibular neuritis[tiab] OR vestibular neuronitis[tiab] OR vestibular syndrome[tiab] OR cerebrovascular[tiab] OR stroke*[tiab] OR cerebellar[tiab] OR hemorrhag*[tiab] OR haemorrhag*[tiab] OR TIA[tiab] OR transient ischemic attack[tiab]) NOT (animals[mh] NOT humans[mh]) AND eng[la] AND 2002:2024[dp] NOT review[pt])

DISAGGREGATED VERSION [to demonstrate overall structure]

((dizz*[tiab] OR vertigo[tiab] OR vestibular[tiab] OR atax*[tiab] NOT “case report”[tiab])

AND

(prodrom*[tiab] OR diagnos*[tiab] OR manifestation*[tiab] OR clinical feature*[tiab] OR symptom*[tiab] OR “physical examination”[mh] OR physical exam*[tiab] OR professional competence[mh] OR “sensitivity and specificity”[tiab] OR “sensitivity and specificity”[mh] OR “reproducibility of results”[mh] OR “observer variation”[mh] OR “diagnostic tests, routine”[mh] OR “decision support techniques”[mh] OR “bayes theorem”[mh] OR dizziness/physiopathology[mh] OR vertigo/physiopathology[mh] OR dizziness/diagnosis[mh] OR vertigo/diagnosis[mh])

AND

(acute peripheral vestibulopathy[tiab] OR labyrin*[tiab] OR vestibular neuritis[tiab] OR vestibular neuronitis[tiab] OR vestibular syndrome[tiab] OR cerebrovascular[tiab] OR stroke*[tiab] OR cerebellar[tiab] OR hemorrhag*[tiab] OR haemorrhag*[tiab] OR TIA[tiab] OR transient ischemic attack[tiab])

NOT (animals[mh] NOT humans[mh])

AND eng[la]

AND 2002:2024[dp]

NOT (review[pt])

### Inclusion and exclusion rules for abstracts & full-text manuscripts

**All gathered literature was subject to title/abstract screening by two independent reviewers (AAT/CM). Abstract review coding rules are provided below. Full-text screening was applied to all citations considered eligible or possibly eligible by at least one reviewer. Two independent reviewers (AAT/ CM) determined whether full-text manuscripts are eligible and, if not, provided a reason for exclusion (see full-text review coding rules below). Differences were resolved by discussion and consensus. CM and AAT completed a hand search of the reference lists of selected articles and published reviews on the topic for additional citations [1, 2]. For citations identified by hand search, the full process was repeated iteratively until no additional manuscripts were found for inclusion. Inter-rater agreement on full-text inclusion was calculated using Cohen’s kappa [3].**

*Abstract Review Coding Rules*

1) Coding status options are “Yes”, “No”, “Maybe”. We will review full text of “Yes” and “Maybe”.

2) Err on the side of “Maybe” if there is doubt about a “No”; this is more conservative.

3) If there is only a title, exclude it only if you feel confident; otherwise code it as “Maybe”.

4) Each "No" should be coded with a reason for exclusion.

5) Reasons for exclusion are listed below 0-7. Go through them in order from 0 to 7 for each abstract, coding the first reason for exclusion only, not multiple reasons for exclusion. Only code "0" for “not English” if you are sure it is “not English”.

6) Two independent raters (AAT/CM) will code reason for exclusion, but we will *not* mandate agreement on exclusion reason at the abstract level.

7) Occasionally an abstract seems inappropriate for another reason. In such cases, code as “other”. There should be few abstracts coded as “other.”

Abstract Reasons for Exclusion

| 0 | not English | manuscript is not in English |
| --- | --- | --- |
| 1 | no data | review paper; no original patient data |
| 2 | not dizziness | no reasonable prospect that the study includes data about dizziness, vertigo or ataxia |
| 3 | not acute | the study does not include data about *acute* (<72 hours) dizziness, vertigo or ataxia obtained during the *acute phase* (<72 hours) of disease |
| 4 | not diagnosis | the study does not include data about clinical diagnostic accuracy about clinical diagnostic accuracy about ataxia in acute central (specifically stroke) or peripheral (specifically vestibular neuritis or BPPV) disorders |
| 5 | <5 cases | fewer than 5 subjects (total participants reported, including cases and controls) |
| 6 | abstract only | only abstract available (from poster presentation or talk at conference) |
| 7 | other | any other reason abstract is not included |

*Full-Text Review Coding Rules*

1) Coding status options are “Yes” or “No”.

2) Each "No" should be coded with a reason for exclusion.

3) Reasons for exclusion are listed below 0-6. Go through them in order from 0 to 6 for each full manuscript, coding the first reason for exclusion only, not multiple reasons for exclusion.

4) Two independent raters (AAT/CM) will code reason for exclusion, and we will mandate agreement on exclusion reason at the manuscript level.

5) Coding differences will be adjudicated or consensus will be developed through dialogue.

Full-Text Reasons for Exclusion

| 0 | not English | manuscript is not in English |
| --- | --- | --- |
| 1 | no data | review paper; no original patient data |
| 2 | not dizziness | the study does not include data about dizziness, vertigo or ataxia |
| 3 | not acute | the study does not include data about *acute* (<72 hours) dizziness, vertigo or ataxia obtained during the *acute phase* (<72 hours) of disease |
| 4 | not diagnosis | the study does not include data about clinical diagnostic accuracy about clinical diagnostic accuracy about ataxia in acute central (specifically stroke) or peripheral (specifically vestibular neuritis or BPPV) disorders |
| 5 | <5 cases | fewer than 5 subjects (total participants reported, including cases and controls) |
| 6 | abstract only | only abstract available (from poster presentation or talk at conference) |

Search Results

Our search identified 7467 unique citations, of which 7361 (98.7%) were excluded at the abstract level (see PRISMA flow chart). We did not demand concordance on reason for abstract exclusion, but, among those abstracts with concordant reasons for exclusion (39.5%, n=2909), the distribution was as follows: 10.9% were **not about** clinical diagnostic accuracy in the assessment of ataxia**; 10.7% were not about vertigo, dizziness or ataxia; 9.6% did not include data about acute (<72h) vertigo, dizziness or ataxia, 5.3% had fewer than 5 subjects studied and 2.9% had no original data.**

We sought to examine 106 full manuscripts (this included 6 articles identified by hand-search). After initial screening, there were a total of 5 disagreements (4.7%) about study inclusion for the two reviewers (**CM** and AAT, kappa=0.85). These differences were resolved by discussion and adjudication by a third reviewer. Overall agreement on reason for exclusion was 96.3%. We demanded concordance on reason for full-text exclusion and resolved differences by discussion.

At the end of our full-text review, 84 were excluded and 22 were considered eligible (see PRISMA flow chart). These eligible studies represented 0.3% of the total (n=7467). Among all full-text manuscripts excluded (79.2%), the distribution of reason for exclusion was as follows: were **not about** clinical diagnostic accuracy in the assessment of ataxia (94.0%), **did not include data about acute (<72h) vertigo, dizziness** or ataxia (2.4%), **had fewer than 5 subjects studied** (2.4%), or contained no original data (1.2%).

For eight studies we attempted to contact the first or corresponding author for additional study information. Four authors responded and provided additional information.

We screened all included manuscripts for other, potentially suitable studies. We identified 6 additional manuscripts, which then were also assessed by both reviewers. Furthermore, we also screened published systematic reviews and consensus papers reporting on a graded gait/truncal instability rating [1, 2].

Assessment of level of evidence

**Level of evidence (LOE) of all studies included after full-text search (n=22) was rated by two independent reviewers (CM/AAT) and disagreements were resolved by discussion. We adhered to the definitions for LOE 1-5 as described in detail below. We did not exclude any studies based on their LOE, but we distinguished between studies with a high LOE (1, 2 and 3) and those with a lower LOE (4 and 5). We identified a total of 7 studies with a high LOE (LOE1=3; LOE2=1; LOE3=3) and 15 studies with a low LOE (LOE4=15; LOE5=0). A quality assessment was performed on all studies using the QUADAS-2 tool. Details are provided in supplementary file 2.**

*Level of evidence (LOE) definitions*

*Level I Evidence:*

Independent, blind comparison of sign or symptom results with a "gold standard" of anatomy, physiology, diagnosis, or prognosis among a large number of consecutive patients suspected of having the target condition.

*Level II Evidence:*

Independent, blind comparison of sign or symptom results with a "gold standard"

among a small number of consecutive patients suspected of having the target condition.

*Level III Evidence:*

Independent, blind comparison of signs and symptoms with a "gold standard" among non-consecutive patients suspected of having the target condition.

*Level IV Evidence:*

Non-independent comparison of signs and symptoms with a "gold standard" among "grab" samples of patients who obviously have the target condition plus, perhaps, normal individuals. --> selection bias. studies restricted to "black and white conditions".

*Level V Evidence:*

Non-independent comparisons of signs and symptoms with a standard of uncertain validity (which may even "incorporate" the sign or symptom result in its definition) among "grab" samples of patients plus, perhaps, normals.

**References**

[1] Shah VP, Oliveira JESL, Farah W, Seisa MO, Balla AK, Christensen A, Farah M, Hasan B, Bellolio F and Murad MH. Diagnostic accuracy of the physical examination in emergency department patients with acute vertigo or dizziness: A systematic review and meta-analysis for GRACE-3. Acad Emerg Med 2023: 30:552-78. doi 10.1111/acem.14630

[2] Kim JS, Newman-Toker DE, Kerber KA, Jahn K, Bertholon P, Waterston J, Lee H, Bisdorff A and Strupp M. Vascular vertigo and dizziness: Diagnostic criteria. J Vestib Res 2022: 32:205-22. doi 10.3233/VES-210169

[3] Cohen J. A coefficient for agreement for nominal scales. Educ Psychol Meas 1960: 20:37-46.
