# Supplementary material for: Systematic Review and Meta-Analysis of the Diagnostic Accuracy of a Graded Gait and Truncal Instability Rating in Acutely Dizzy and Ataxic Patients
Source: Cerebellum. 2024 Jul 11;23(6):2244–56. doi: 10.1007/s12311-024-01718-6 (PMC11585515; doi:10.1007/s12311-024-01718-6)
Supplement: Supplementary file 2 — Additional file 2. [file 12311_2024_1718_MOESM2_ESM.docx]

**Supplementary file 2 - QUADAS-2 assessment of included studies**

For included studies, two independent raters (CM/AAT) assessed the risk of bias or applicability concerns using QUADAS-2 [1] tailored study criteria, resolving disagreements by discussion. The QUADAS-2 tool for quality rating of diagnostic accuracy studies consists of four core domains (patient selection, index test, reference standard, and flow and timing) [1]. Risk of bias is assessed for all four domains, and applicability is assessed for the first three domains. Thus, seven items per study are assessed to rate quality of evidence (see Table S1). For each item, pre-specified conditions must be met to qualify for “low risk” of bias. The tool’s authors recommend review-specific tailoring of the rating criteria and process [1], which we did for all seven criteria (Box 1).

Overall, few studies were low risk of bias and low risk of applicability concerns in all items [2-5], whereas most studies (n=18) demonstrated high risk of bias for at least one item. Most frequently, there was a high risk of bias regarding patient selection (n=10 studies).

*eBox 1. Tailored QUADAS-2 rating criteria for key domains of bias or applicability concerns (modified after [6])*

- Patient selection (bias): Studies were rated as *low* risk if the population was well-described and unbiased. Studies were rated as *unclear* if patients were drawn from a prospective patient registry without further explanation of original selection. Studies were rated as *high* risk if patients were chosen in non-consecutive/non-random fashion or if they studied a known clinical or demographic subgroup in whom test properties of bedside findings were likely overstated (e.g., sensitivity of neurologic findings for stroke if only patients with such findings were investigated) or understated (e.g., eye movement exams applied to incorrect subpopulations of dizziness/vertigo, such as head impulse testing applied to patients with episodic, positional vertigo).
- Patient selection (applicability concerns): Studies were rated as *low* risk if they used clear, accepted definitions for AVS and/or positional EVS (eBox 2) [7, 8]. Studies were rated as *unclear* if patients were classified but without clear definitions (e.g., said to have AVS, but without defining AVS directly or referencing previously-published criteria). Studies were rated as *high* risk if patients were classified with clear definitions that did not align with accepted definitions.
- Index test (bias): Studies were rated as *low* risk if bedside (index) tests were performed by subspecialists (neuro-ophthalmology, neuro-otology, or other clinicians routinely using vestibular tests [e.g., vestibular physical therapists, vestibular lab technicians]) or trained specialists (e.g., neurologists) or generalists (e.g., ED physicians) where rater skills were shown to be accurate and reliable. Studies were rated *unclear* if tests were performed by untrained specialists (general neurologists or otolaryngologists); specialty trainees (e.g., neurology or otolaryngology residents); if the training of those who performed the bedside examinations was either mixed (e.g., specialists and ED physicians); or if training or masking was not specifically reported. Studies were rated as having *high* risk if more than 20% of tests were performed by generalists who were untrained or whose test performance was of low quality or not assessed.
- Index test (applicability concerns): Studies were rated as *low* risk if bedside test techniques were described in sufficient detail to match accepted standards. Studies were rated as having *high* risk if bedside test techniques were described or demonstrated (e.g., by video) to not match accepted standards or were inconsistently applied. Studies were rated as *unclear* if bedside techniques were not described sufficiently.
- Reference standard (bias): The risk of bias in the reference standard was determined using rules specific to each diagnosis (see eBox 3).
- Reference standard (applicability concerns): Studies were rated as *low* risk if standard diagnostic criteria were used to define stroke and/or peripheral vestibular disorders, and these criteria were compatible with existing consensus definitions and guidelines [9-14]. Studies were rated as *unclear* if definitions were not reported. Studies were rated as *high* risk if non-standard definitions were applied.
- Flow and timing (bias): Studies were rated as *low* risk if over 80% of patients underwent the same (or an equivalently valid) reference standard in a timely manner. Studies were rated as *unclear* if details were not reported. Studies were rated as *high* risk if the reference standard varied or timing was not appropriate (e.g., if an MRI reference standard was too early or too late, lowering its sensitivity) in more than 20%.

Abbreviations: AVS – acute vestibular syndrome; ED – emergency department; EVS – episodic vestibular syndrome.

*eBox 2. Consensus (ICVD) Definitions for Vestibular Syndromes (i.e., dizziness/vertigo subpopulations)*

- Acute Vestibular Syndrome (AVS): A clinical syndrome of acute-onset, continuous vertigo, dizziness, or unsteadiness lasting days to weeks, and generally including features suggestive of new, ongoing vestibular system dysfunction (e.g., vomiting, nystagmus, postural instability).
- Episodic Vestibular Syndrome (EVS): A clinical syndrome of transient vertigo, dizziness, or unsteadiness lasting seconds to hours, occasionally days, and generally including features suggestive of temporary, short-lived vestibular system dysfunction (e.g., nausea, nystagmus, sudden falls).
- Chronic Vestibular Syndrome (CVS): A clinical syndrome of chronic vertigo, dizziness, or unsteadiness lasting months to years and generally including features suggestive of persistent vestibular system dysfunction (e.g., oscillopsia, nystagmus, gait unsteadiness).

Abbreviations: ICVD – International Classification of Vestibular Disorders

*eBox 3. Valid reference standards for central and peripheral vestibular disorders with risk of bias (modified after [6])*

- Stroke: Strokes must have been confirmed by neuroimaging to be considered valid. The type of imaging and imaging result determined the QUADAS-2 risk of bias on reference standard. To be considered *low* risk, strokes had to be clinically deemed causal and confirmed to be new/acute lesions by neuroimaging. To be considered causal, rather than incidental, strokes must have been found in a brain region appropriate to the clinical syndrome (brainstem, cerebellum, thalamus, or parieto-insular region if hemispheric). To be confirmed new/acute by neuroimaging, strokes must have been demonstrated by (1) MRI-DWI showing restricted diffusion; (2) clearly new lesion evident by post-onset serial CT or MRI (e.g., no lesion on day #1 but present on day #3); or (3) acute brain hemorrhages or large ischemic strokes clearly evident by CT (e.g., based on demarcation, attenuation, and/or mass effect). Studies that demonstrably fell short of these requirements were rated as *high* risk, whereas studies not providing sufficient detail on neuroimaging were considered of *unclear* risk. If testing protocols were non-uniform across patients, no more than 20% of patients could be in a higher risk category to still maintain the lower risk category for QUADAS-2 status.
- Vestibular neuritis (without hearing loss) or labyrinthitis (with hearing loss): Patients must have had an AVS clinical presentation, a spontaneous or gaze-evoked nystagmus consistent with Alexander’s law, evidence of a unilateral vestibular deficit on quantitative physiologic testing (caloric or quantified head impulse testing), and either no associated (new) neurologic deficits to suggest a central nervous system disorder or negative neuroimaging (by CT or MRI) to be considered valid. The type of imaging and time window from onset of AVS symptoms determined the QUADAS-2 risk of bias on reference standard: *low* risk (MRI-DWI 48 hours to 14 days, inclusive), *unclear* risk (MRI-DWI 0-48 hours), *high* risk (otherwise). If the imaging type or time windows were non-uniform across patients, no more than 20% of patients could be in a higher risk category to still maintain the lower risk category for QUADAS-2 status.

Abbreviations: AVS – acute vestibular syndrome; DWI – diffusion weighted imaging; EVS – episodic vestibular syndrome; ICVD – International Classification of Vestibular Disorders

**Table S2-1 – QUADAS2 risk of bias assessment of all studies included after full-text ratings (n=22)**

|  |  | **Risk of Bias** | | | | **Applicability Concerns** | | |
| --- | --- | --- | --- | --- | --- | --- | --- | --- |
| **Citation** | **Sample size (n)** | **Patient Selection** | **Index Test** | **Reference Standard** | **Flow & Timing** | **Patient Selection** | **Index Test** | **Reference Standard** |
| Carmona et al. 2016 [15] | 114 | high risk | low risk | low risk | low risk | low risk | low risk | low risk |
| Carmona et al. 2023 [16] | 95 | high risk | low risk | high risk | low risk | unclear | unclear | low risk |
| Casani et al. 2013 [17] | 11 | high risk | low risk | low risk | low risk | low risk | low risk | low risk |
| Chen et al. 2011 [18] | 24 | low risk | low risk | low risk | low risk | low risk | unclear | unclear |
| Chen et al. 2014 [19] | 53 | high risk | low risk | low risk | low risk | low risk | unclear | unclear |
| Choi et al. 2014 [20] | 34 | low risk | low risk | low risk | low risk | unclear | low risk | low risk |
| Choi et al. 2015 [21] | 8 | high risk | low risk | low risk | low risk | unclear | low risk | low risk |
| Choi et al. 2017 [22] | 23 | low risk | low risk | unclear | low risk | low risk | unclear | low risk |
| Choi et al. 2018 [23] | 29 | low risk | low risk | low risk | low risk | unclear | unclear | low risk |
| Kattah et al. 2009 [4] | 101 | low risk | low risk | low risk | low risk | low risk | low risk | low risk |
| Kattah et al. 2022 [24] | 52 | high risk | low risk | low risk | low risk | unclear | low risk | low risk |
| Kim 2003 [25] | 130 | high risk | low risk | low risk | low risk | unclear | unclear | unclear |
| Kmetonyova et al. 2022 [26] | 119 | low risk | low risk | high risk | low risk | low risk | unclear | low risk |
| Lee et al. 2006 [5] | 25 | low risk | low risk | low risk | low risk | low risk | low risk | low risk |
| Liu et al. 2024 [3] | 121 | low risk | low risk | low risk | low risk | low risk | low risk | low risk |
| Moon et al. 2009 [27] | 8 | high risk | low risk | low risk | low risk | unclear | unclear | unclear |
| Nam et al. 2021 [28] | 34 | high risk | low risk | high risk | low risk | low risk | low risk | low risk |
| Newman-Toker et al. 2013 [2] | 191 | low risk | low risk | low risk | low risk | low risk | low risk | low risk |
| Ogawa et al. 2017 [29] | 7 | high risk | unclear | low risk | low risk | unclear | unclear | unclear |
| Vanni et al. 2014 [30] | 98 | low risk | low risk | high risk | low risk | low risk | low risk | low risk |
| Vanni et al. 2017 [31] | 352 | low risk | low risk | high risk | low risk | low risk | low risk | low risk |
| Ye et al. 2010 [32] | 66 | low risk | low risk | low risk | low risk | unclear | unclear | unclear |

**References**

[1] Whiting PF, Rutjes AW, Westwood ME, Mallett S, Deeks JJ, Reitsma JB, Leeflang MM, Sterne JA, Bossuyt PM and Group Q-. QUADAS-2: a revised tool for the quality assessment of diagnostic accuracy studies. Ann Intern Med 2011: 155:529-36. doi 10.7326/0003-4819-155-8-201110180-00009

[2] Newman-Toker DE, Kerber KA, Hsieh YH, Pula JH, Omron R, Saber Tehrani AS, Mantokoudis G, Hanley DF, Zee DS and Kattah JC. HINTS outperforms ABCD2 to screen for stroke in acute continuous vertigo and dizziness. Acad Emerg Med 2013: 20:986-96. doi 10.1111/acem.12223

[3] Liu X, Li Z, Ju Y and Zhao X. Application of bedside HINTS, ABCD(2) score and truncal ataxia to differentiate cerebellar-brainstem stroke from vestibular neuritis in the emergency room. Stroke Vasc Neurol 2024. doi 10.1136/svn-2023-002779

[4] Kattah JC, Talkad AV, Wang DZ, Hsieh YH and Newman-Toker DE. HINTS to diagnose stroke in the acute vestibular syndrome: three-step bedside oculomotor examination more sensitive than early MRI diffusion-weighted imaging. Stroke 2009: 40:3504-10. doi 10.1161/STROKEAHA.109.551234

[5] Lee H, Sohn SI, Cho YW, Lee SR, Ahn BH, Park BR and Baloh RW. Cerebellar infarction presenting isolated vertigo: frequency and vascular topographical patterns. Neurology 2006: 67:1178-83. doi 10.1212/01.wnl.0000238500.02302.b4

[6] Tarnutzer AA, Gold D, Wang Z, Robinson KA, Kattah JC, Mantokoudis G, Saber Tehrani AS, Zee DS, Edlow JA and Newman-Toker DE. Impact of clinician training background and stroke location on bedside diagnostic accuracy in the acute vestibular syndrome -a meta-analysis. Ann Neurol 2023. doi 10.1002/ana.26661

Newman-Toker D. Vestibular syndrome definitions for the International Classification of Vestibular Disorders. Bárány Classification Committee Meeting on March 10-11, 2017*.* Berlin, Germany; 2017.

[8] Newman-Toker DE and Edlow JA. TiTrATE: A Novel, Evidence-Based Approach to Diagnosing Acute Dizziness and Vertigo. Neurol Clin 2015: 33:577-99, viii. doi 10.1016/j.ncl.2015.04.011

[9] Bhattacharyya N, Gubbels SP, Schwartz SR, Edlow JA, El-Kashlan H, Fife T, Holmberg JM, Mahoney K, Hollingsworth DB, Roberts R, Seidman MD, Steiner RW, Do BT, Voelker CC, Waguespack RW and Corrigan MD. Clinical Practice Guideline: Benign Paroxysmal Positional Vertigo (Update). Otolaryngol Head Neck Surg 2017: 156:S1-S47. doi 10.1177/0194599816689667

[10] Easton JD, Saver JL, Albers GW, Alberts MJ, Chaturvedi S, Feldmann E, Hatsukami TS, Higashida RT, Johnston SC, Kidwell CS, Lutsep HL, Miller E, Sacco RL, American Heart A, American Stroke Association Stroke C, Council on Cardiovascular S, Anesthesia, Council on Cardiovascular R, Intervention, Council on Cardiovascular N and Interdisciplinary Council on Peripheral Vascular D. Definition and evaluation of transient ischemic attack: a scientific statement for healthcare professionals from the American Heart Association/American Stroke Association Stroke Council; Council on Cardiovascular Surgery and Anesthesia; Council on Cardiovascular Radiology and Intervention; Council on Cardiovascular Nursing; and the Interdisciplinary Council on Peripheral Vascular Disease. The American Academy of Neurology affirms the value of this statement as an educational tool for neurologists. Stroke 2009: 40:2276-93. doi 10.1161/STROKEAHA.108.192218

[11] Fife TD, Iverson DJ, Lempert T, Furman JM, Baloh RW, Tusa RJ, Hain TC, Herdman S, Morrow MJ, Gronseth GS and Quality Standards Subcommittee AAoN. Practice parameter: therapies for benign paroxysmal positional vertigo (an evidence-based review): report of the Quality Standards Subcommittee of the American Academy of Neurology. Neurology 2008: 70:2067-74. doi 10.1212/01.wnl.0000313378.77444.ac

[12] von Brevern M, Bertholon P, Brandt T, Fife T, Imai T, Nuti D and Newman-Toker D. Benign paroxysmal positional vertigo: Diagnostic criteria. J Vestib Res 2015: 25:105-17. doi 10.3233/VES-150553

[13] Lopez-Escamez JA, Carey J, Chung WH, Goebel JA, Magnusson M, Mandala M, Newman-Toker DE, Strupp M, Suzuki M, Trabalzini F, Bisdorff A, Classification Committee of the Barany S, Japan Society for Equilibrium R, European Academy of O, Neurotology, Equilibrium Committee of the American Academy of O-H, Neck S and Korean Balance S. Diagnostic criteria for Meniere's disease. J Vestib Res 2015: 25:1-7. doi 10.3233/VES-150549

[14] Lempert T, Olesen J, Furman J, Waterston J, Seemungal B, Carey J, Bisdorff A, Versino M, Evers S, Kheradmand A and Newman-Toker D. Vestibular migraine: Diagnostic criteria1. J Vestib Res 2022: 32:1-6. doi 10.3233/VES-201644

[15] Carmona S, Martinez C, Zalazar G, Moro M, Batuecas-Caletrio A, Luis L and Gordon C. The Diagnostic Accuracy of Truncal Ataxia and HINTS as Cardinal Signs for Acute Vestibular Syndrome. Front Neurol 2016: 7:125. doi 10.3389/fneur.2016.00125

[16] Carmona S, Martinez C, Zalazar G, Koohi N and Kaski D. Acute truncal ataxia without nystagmus in patients with acute vertigo. Eur J Neurol 2023: 30:1785-90. doi 10.1111/ene.15729

[17] Casani AP, Dallan I, Cerchiai N, Lenzi R, Cosottini M and Sellari-Franceschini S. Cerebellar infarctions mimicking acute peripheral vertigo: how to avoid misdiagnosis? Otolaryngol Head Neck Surg 2013: 148:475-81. doi 10.1177/0194599812472614

[18] Chen L, Lee W, Chambers BR and Dewey HM. Diagnostic accuracy of acute vestibular syndrome at the bedside in a stroke unit. J Neurol 2011: 258:855-61. doi 10.1007/s00415-010-5853-4

[19] Chen L, Todd M, Halmagyi GM and Aw S. Head impulse gain and saccade analysis in pontine-cerebellar stroke and vestibular neuritis. Neurology 2014: 83:1513-22. doi 10.1212/WNL.0000000000000906

[20] Choi JH, Kim HW, Choi KD, Kim MJ, Choi YR, Cho HJ, Sung SM, Kim HJ, Kim JS and Jung DS. Isolated vestibular syndrome in posterior circulation stroke: Frequency and involved structures. Neurol Clin Pract 2014: 4:410-8. doi 10.1212/CPJ.0000000000000028

[21] Choi JH, Seo JD, Choi YR, Kim MJ, Kim HJ, Kim JS and Choi KD. Inferior cerebellar peduncular lesion causes a distinct vestibular syndrome. Eur J Neurol 2015: 22:1062-7. doi 10.1111/ene.12705

[22] Choi JH, Park MG, Choi SY, Park KP, Baik SK, Kim JS and Choi KD. Acute Transient Vestibular Syndrome: Prevalence of Stroke and Efficacy of Bedside Evaluation. Stroke 2017: 48:556-62. doi 10.1161/STROKEAHA.116.015507

[23] Choi JH, Oh EH, Park MG, Baik SK, Cho HJ, Choi SY, Lee TH, Kim JS and Choi KD. Early MRI-negative posterior circulation stroke presenting as acute dizziness. J Neurol 2018: 265:2993-3000. doi 10.1007/s00415-018-9097-z

[24] Kattah JC, Martinez C, Zalazar G, Batuecas A, Lemos J and Carmona S. Role of incubitus truncal ataxia, and equivalent standing grade 3 ataxia in the diagnosis of central acute vestibular syndrome. J Neurol Sci 2022: 441:120374. doi 10.1016/j.jns.2022.120374

[25] Kim JS. Pure lateral medullary infarction: clinical-radiological correlation of 130 acute, consecutive patients. Brain 2003: 126:1864-72. doi 10.1093/brain/awg169

[26] Kmetonyova S, Paulasova Schwabova J, Sramkova T, Dankova M, Olserova A, Petrzalka M, Tomek A and Jerabek J. Posterior circulation stroke diagnosis in unselected group of acutely dizzy patients. Clin Neurol Neurosurg 2023: 224:107541. doi 10.1016/j.clineuro.2022.107541

[27] Moon IS, Kim JS, Choi KD, Kim MJ, Oh SY, Lee H, Lee HS and Park SH. Isolated nodular infarction. Stroke 2009: 40:487-91. doi 10.1161/STROKEAHA.108.527762

[28] Nam GS, Shin HJ, Kang JJ, Lee NR and Oh SY. Clinical Implication of Corrective Saccades in the Video Head Impulse Test for the Diagnosis of Posterior Inferior Cerebellar Artery Infarction. Front Neurol 2021: 12:605040. doi 10.3389/fneur.2021.605040

[29] Ogawa K, Suzuki Y, Takahashi K, Akimoto T, Kamei S and Soma M. Clinical Study of Seven Patients with Infarction in Territories of the Anterior Inferior Cerebellar Artery. J Stroke Cerebrovasc Dis 2017: 26:574-81. doi 10.1016/j.jstrokecerebrovasdis.2016.11.118

[30] Vanni S, Pecci R, Casati C, Moroni F, Risso M, Ottaviani M, Nazerian P, Grifoni S and Vannucchi P. STANDING, a four-step bedside algorithm for differential diagnosis of acute vertigo in the Emergency Department. Acta Otorhinolaryngol Ital 2014: 34:419-26.

[31] Vanni S, Pecci R, Edlow JA, Nazerian P, Santimone R, Pepe G, Moretti M, Pavellini A, Caviglioli C, Casula C, Bigiarini S, Vannucchi P and Grifoni S. Differential Diagnosis of Vertigo in the Emergency Department: A Prospective Validation Study of the STANDING Algorithm. Front Neurol 2017: 8:590. doi 10.3389/fneur.2017.00590

[32] Ye BS, Kim YD, Nam HS, Lee HS, Nam CM and Heo JH. Clinical manifestations of cerebellar infarction according to specific lobular involvement. Cerebellum 2010: 9:571-9. doi 10.1007/s12311-010-0200-y
