# Supplementary material for: Systematic Review and Meta-Analysis of the Diagnostic Accuracy of a Graded Gait and Truncal Instability Rating in Acutely Dizzy and Ataxic Patients
Source: Cerebellum. 2024 Jul 11;23(6):2244–56. doi: 10.1007/s12311-024-01718-6 (PMC11585515; doi:10.1007/s12311-024-01718-6)
Supplement: Supplementary file 3 — Additional file 3. [file 12311_2024_1718_MOESM3_ESM.docx]

# **Supplementary file 3 – additional tables**

## **Table S3-1: Studies included in the meta-analysis (n=18)**

| **Author (citation)** | **Year** | **Total Dizziness Sample (studied, % females)** | **Definition of total sample** | **Population (Study Focus)** | **Setting (country)** | **Bedside testing performed by** | **LOE*** | **Data collection (source, analysis)** | **Mean age [years] (standard deviation, range)** | **Special comments** |
| --- | --- | --- | --- | --- | --- | --- | --- | --- | --- | --- |
| Carmona et al. [1] | 2016 | 1218 (114, 58%) | ED presentation due to “dizziness” | **Isolated AVS** (clinical findings: HINTS vs. truncal ataxia) | ED, single reference hospital (Argentina) | Trained neurology residents supervised by experienced neuro-otologists | 3 | Retrospective (ED population, cross-sectional) | cAVS: 57.9 (11, NR)  pAVS: 43.3 (14.9, NR) |  |
| Carmona et al. [2] | 2023 | NR (95, 47%) | NR | **AVS**  (diagnosis, clinical features, imaging) | Four academic hospitals (Argentina, UK) | Experienced neuro-otologists | 4 | Retrospective (ED/hospital admission population, cross-sectional) | cAVS and pAVS pooled (Argentina): 58 (8.3, 30-80)  cAVS and pAVS pooled (UK): 57 (16.5, 25-83) |  |
| Casani et al. [3] | 2013 | NR (11, 55%) | Patients presenting to the ED with isolated acute spontaneous vertigo (2007-2011) | **Central isolated AVS initially misdiagnosed as peripheral AVS** | ED, single reference hospital (Italy) | Experienced neuro-otologists | 4 | Retrospective (ED/hospital admission population, cross-sectional) | cAVS: 62.5 (11, 47-80) |  |
| Chen et al. [4] | 2011 | 36 (24, 37%) | ED presentation with „acute prolonged rotatory vertigo associated with nausea and/or vomiting, without other brainstem signs“ | **Isolated AVS** (iAVS) (clinical findings) | ED, single tertiary hospital (Australia) | Neurologists with 4 hours of training in neuro-otology | 2 | Prospective (ED population, cross-sectional) | 64·0 (13, 42-83) |  |
| Chen et al. [5] | 2014 | NR (53, 34%) | Patients presenting to the ED with acute prolonged spontaneous vertigo (>24h) and gait imbalance | **AVS**  (diagnosis, clinical features, imaging) | ED, single tertiary hospital (Australia) | Experienced neuro-otologists | 4 | Prospective (ED population, cross-sectional) | cAVS: 58.5 (15.8, 24-82)  pAVS: 59.2 (14.5, 37-85) |  |
| Choi et al. [6] | 2014 | 132 (34, 24%) | Patients with confirmed posterior-circulation stroke | **Central isolated AVS**  (imaging) | Single academic hospital (South Korea) | Experienced neuro-otologists | 4 | Prospective (ED population, cross-sectional) | cAVS: 64.6 (12.4, 30-85) |  |
| Choi et al. [7] | 2015 | NR (8, 63%) | Patients presenting to the dizzy clinic with acute prolonged vertigo | **Central AVS due to isolated unilateral lesions of the inferior cerebellar peduncle**  (clinical findings, laboratory testing) | Single academic hospital (South Korea) | Experienced neuro-otologists | 4 | Prospective (ED population, cross-sectional) | cAVS: 53.6 (21.4, 18-83) | Delay from symptom onset to clinical examination not reported, however, symptoms were ongoing in all patients. |
| Choi et al. [8] | 2017 | 194 (23, 22%) | Patients with acute transient vestibular syndrome | **Acute transient vestibular syndrome of central origin** | Single academic hospital (South Korea) | Experienced neuro-otologists | 2 | Prospective (ED/hospital admission population, cross-sectional) | cAVS: 59 (16, NR) | Note that HINTS were applied only on those patients that had ongoing vestibular symptoms when presenting. Only this subset of patients was included here. |
| Choi et al. [9] | 2018 | 1846 (29, 38%) | Patients with acute ischemic stroke or a transient-ischemic attack (2010-2017) | Stroke patients with initially MRI-DWI negative PCS and acute vertigo | Two academic hospitals (South Korea) | Experienced neuro-otologists | 4 | Prospective (ED population, cross-sectional) | cAVS: 62.0 (13.8, 31-89) | Six patients were previously reported by Choi et al. 2014. They were excluded here to avoid duplicity |
| Kattah et al. [10] | 2022 | NR (52, 52%) | NR | **Isolated AVS**  (diagnosis, clinical features, imaging) | Four academic hospitals (Argentina, Spain, Portugal) | Experienced neuro-otologists | 3 | Prospective (ED/hospital admission population, cross-sectional) | cAVS: 62 (10, NR)  pAVS: 50 (13, NR) |  |
| Kim [11] | 2003 | 222 (130, 44%) | Patients with suspected lateral medullary infarction | **Patients with confirmed lateral medullary infarction** (imaging) | Single academic hospital (South Korea) | Neurologist, not further specified | 4 | Prospective (ED population, cross-sectional) | cAVS: 57 (11.9, 28-84) | Note that not all patients presented with acute vertigo (57%) or gait ataxia (92%). However, no single subject data was available to exclude those patients not presenting with vertigo or gait ataxia.  Note also that the majority of patients were seen within 3 days after symptom onset, however, more detailed numbers are missing. |
| Lee et al. [12] | 2006 | 240 (25, 44%) | Patients with isolated cerebellar infarction (2000-2005) | **Isolated AVS and diagnosis of cerebellar infarction** (clinical findings and audio-vestibular testing, vascular territory) | Single academic hospital (South Korea) | Neurologists, not further specified | 4 | Likely prospective (ED population, cross-sectional) | cAVS: 63.7 (10.6, 37-80) |  |
| Liu et al. [13] | 2024 |  | NR | **AVS**  (diagnosis, clinical features, imaging) | Single academic hospital (China) | Experienced neuro-otologists | 1 | Prospective (ED population, cross-sectional) | cAVS: 61.0 (52.3-68.8)  pAVS: 47 (36.0-59.9)  (median, IQR [inter-quartile range]) | Definition of HINTS was slightly modified such as that vertical or isolated torsional nystagmus was considered a central-type nystagmus as well (besides gaze-evoked nystagmus) |
| Moon et al. [14] | 2009 | (8, 38%) | NR | **AVS and diagnosis of isolated infarction of the cerebellar nodulus** (clinical features, audio-vestibular findings) | Six academic hospitals (South Korea) | Neurologists, not further specified | 4 | Prospective (ED/hospital admission population from stroke registry, consecutive case series) | cAVS: 60.1 (12.4, 37-75) | Delay from symptom onset to clinical examination not reported, however, symptoms were ongoing in all patients. |
| Nam et al. [15] | 2021 | NR (34, 41%) | NR | **AVS**  (imaging) | Single academic hospital (South Korea) | Experienced neuro-otologists | 4 | Prospective (ED/hospital admission population, cross-sectional) | cAVS: 65.8 (13.7, NR)  pAVS: 56.5 (16, NR) | Delay from symptom onset to clinical examination not reported. Inclusion possible for symptom duration less than 7 days. |
| Newman-Toker et al. [16]  Preliminary report in Kattah et al. 2009 [17] | 2013 | 191 (191, 40%) | “Patients with at least 1 hour of acute, persistent, continuous vertigo or dizziness with spontaneous or gaze-evoked nystagmus, plus nausea or vomiting, head motion intolerance, and new gait unsteadiness (i.e., AVS), presenting within 1 week of symptom onset“ | **AVS and > 1 stroke risk factor** (diagnosis, clinical features, imaging) | Single academic hospital (USA) | Experienced neuro-otologist | 1 | Prospective (ED/hospital admission population, cross-sectional) | 61.0 (NR, 18-92) | Patients with initial negative cMRI underwent repeat cMRI for unexplained signs suggesting brainstem location. Based on the dataset provided by the corresponding author, we included one additional patient that was not considered in the original publication. |
| Ogawa et al. [18] | 2017 | NR (7, 14%) | NR | **Confirmed AICA stroke and gait ataxia** | Single academic hospital (Japan) | Neurologists, not further specified | 4 | Retrospective (ED/hospital admission population, cross-sectional) | cAVS: 55.1 (13.2, 32-72) | MRI was obtained between 15 hours and 5 days after symptom onset. Likely clinical assessment was obtained in most patients within 72 hours, but detailed numbers are missing. |
| Ye et al. [19] | 2010 | 3821 (66, 24%) | All patients with confirmed stroke between Jan 1999 and Jul 2008 | **AVS** (Patients with confirmed cerebellar stroke and acute vertigo / lateropulsion) | Single academic hospital (South Korea) | Neurologists, not further specified | 4 | Retrospective (ED/hospital admission population, cross-sectional) | Median age reported only (62 years, range 20-83 years) | Initial assessment including MRI was obtained 1.2 days after symptom onset (median value) |

* Level of evidence was determined as defined by (criteria may be found in supplementary file 1).

Abbreviations: AICA = anterior inferior cerebellar artery; AVS = acute vestibular syndrome (vertigo, nystagmus, nausea/vomiting, head-motion intolerance, unsteady gait lasting ≥24h); cCT = cranial computer tomography; MRI-DWI = cranial magnetic resonance imaging with diffusion-weighted imaging; ED = emergency department; NA = not applicable; NR = not reported; PCS = posterior circulation stroke.

## **Table S3-2: diagnostic accuracy of selected bedside diagnostic tests in patients with acute vestibular syndrome**

| **Supplementary table S3-2 – diagnostic accuracy of selected bedside diagnostic tests in patients with acute vestibular syndrome** | | | | | | | | | | | | | |
| --- | --- | --- | --- | --- | --- | --- | --- | --- | --- | --- | --- | --- | --- |
|  |  |  |  |  |  |  |  |  |  |  |  | **Cochran’s Q-Test of heterogeneity** | |
| **Bedside diagnostic tests** | **n[studies]** | **n[subjects]** | | **Sensitivity (%)** | **Specificity (%)** | **95% CI Sensitivity** | **95% CI Specificity** | **LR-** | **LR+** | **95% CI LR-** | **95% CI LR+** | **Sensitivity**  **p value** | **Specificity**  **p value** |
|  | **Total, pAVS, cAVS** | **pAVS** | **cAVS** |  |  |  |  |  |  |  |  |  |  |
|  |  |  |  |  |  |  |  |  |  |  |  |  |  |
| **HINTS plus** [1, 4, 5, 7, 16] | 5, 4, 5 | 172 | 218 | 98.9 | 95.1* | 97.5-100.0 | 90.0-100.0* | 0.03 | 12.50 | 0.01-0.11 | 3.05-51.20 | p=0.697 | p=0.075 |
| **HINTS** [1, 4-7, 9, 13, 16] | 8, 5, 8 | 241 | 333 | 96.8 | 97.6 | 94.8-98.8 | 95.3-99.9 | 0.07 | 17.83 | 0.03-0.14 | 7.63-41.64 | p=0.381 | p=0.263 |
| **Normal hHIT** (all cAVS) [1, 3-7, 9, 12-16] | 12, 6, 12 | 258 | 392 | 91.4 | 99.1 | 87.4-95.4 | 98.0-100.0 | 0.17 | 31.82 | 0.13-0.23 | 9.91-102.20 | p=0.018 | p=0.775 |
| **Direction-changing nystagmus** † [1, 3-7, 9, 12, 13, 15, 16, 18] | 12, 6, 12 | 258 | 392 | 26.5 | 99.2 | 17.2-35.8 | 98.1-100.0 | 0.71 | 22.05 | 0.62-0.83 | 7.04-69.08 | p<0.001 | p=0.977 |
| **Skew deviation** [1, 4-7, 9, 11, 13, 15, 16] | 10, 6, 10 | 258 | 479 | 20.2 | 98.0 | 10.1-30.2 | 96.2-99.8 | 0.82 | 6.86 | 0.72-0.93 | 3.53-13.32 | p<0.001 | p=0.379 |
| **No spontaneous horizontal nystagmus** [1-7, 9, 10, 12, 14-16, 18, 19] | 15, 7, 15 | 189 | 389 | 46.7 | 98.8 | 35.1-58.3 | 97.3-100.0 | 0.55 | 14.52 | 0.45-0.68 | 3.83-55.12 | p<0.001 | p=0.646 |
| **Truncal instability (grade 1/2/3)** ‡ [1-3, 5-7, 9-14, 16, 18, 19] | 15, 6, 15 | 285 | 658 | 91.8 | 25.7 | 88.0-95.6 | 0.0-73.9 | 0.25 | 1.07 | 0.19-0.33 | 0.98-1.16 | p<0.001 | p<0.001 |
| ***PICA-stroke vs. APV only*** [1, 2, 5, 6, 10, 12, 14, 16] | *8, 5, 8 §* | *216* | *220* | *95.7* | *22.4 §* | *92.2-99.2* | *0.0-76.4 §* | *0.19* | *1.02 §* | *0.07-0.51* | *0.93-1.11 §* | p=0.058 | p<0.001 |
| ***AICA-stroke vs. APV only*** [1, 2, 5, 6, 10, 12, 16, 18] | *8, 5, 8 §* | *216* | *58* | *93.8* | *22.4 §* | *88.1-99.5* | *0.0-76.4 §* | *0.41* | *1.00 §* | *0.19-0.90* | *0.87-1.14 §* | p=0.827 | p<0.001 |
| **Truncal instability (grade 2/3)** ‡ [1-3, 5-7, 9, 10, 12-14, 16, 18, 19] | 14, 6, 14 | 285 | 528 | 70.8 | 82.7 | 59.3-82.3 | 71.6-93.8 | 0.30 | 4.47 | 0.19-0.46 | 2.44-8.18 | p<0.001 | p<0.001 |
| ***PICA-stroke vs. APV only*** [1, 2, 5, 6, 10, 12, 14, 16] | *8, 5, 8 §* | *216* | *220* | *83.5* | *79.2 §* | *73.8-93.2* | *61.4-97.0 §* | *0.23* | *3.49 §* | *0.10-0.52* | *1.90-6.39 §* | p<0.001 | p<0.001 |
| ***AICA-stroke vs. APV only*** [1, 2, 5, 6, 10, 12, 16, 18] | *8, 5, 8 §* | *216* | *58* | *77.4* | *79.2 §* | *63.1-91.6* | *61.4-97.0 §* | *0.40* | *3.41 §* | *0.24-0.65* | *2.05-5.66 §* | p=0.019 | p<0.001 |
| **Truncal instability (grade 3)** \|\| [1-16, 18, 19] | 17, 7, 17 | 247 | 656 | 44.0 | 99.1 | 34.3-53.7 | 98.0-100.0 | 0.55 | 29.61 | 0.43-0.71 | 10.78-81.32 | p<0.001 | p=0.994 |
| ***PICA-stroke vs. APV only*** [1, 2, 5, 6, 10, 12, 14-16] | 9, 5, 9 § | 247 | 237 | *52.2* | 99.0§ | *41.6-62.8* | 97.8-100.0 § | *0.50* | 34.08 § | *0.39-0.65* | 10.93-106.24 § | p=0.005 | p=0.982 |
| ***AICA-stroke vs. APV only*** [1, 2, 5, 6, 10 , 12, 16, 18] | 8, 5, 8 § | 247 | 58 | *62.0* | 99.1§ | *41.7-82.3* | 97.8-100.0 § | *0.47* | 43.37 § | *0.29-0.77* | 12.35-152.36 § | p=0.001 | p=0.975 |
| **Truncal instability and/or central-type nystagmus (grade 1/2/3)** ‡,**¶, **** [3, 6, 9, 10, 12, 14, 16, 18] | 8, 3, 8 | 93 | 263 | 88.2 | 69.9 | 84.3-92.1 | 60.6-79.2 | 0.17 | 2.93 | 0.12-0.24 | 2.14-4.01 | NA** | NA** |
| **Truncal instability and/or central-type nystagmus (grade 2/3)** ‡,**¶, **** [3, 6, 9, 10, 12, 14, 16, 18] | 8, 3, 8 | 93 | 263 | 77.2 | 90.3 | 72.1-82.3 | 84.3-96.3 | 0.25 | 7.98 | 0.20-0.32 | 4.27-14.89 | NA** | NA** |
| **Truncal instability and/or central-type nystagmus (grade 3)** \|\|,**¶, **** [3, 4, 6, 9, 10, 12, 14, 16, 18] | 9, 3, 9 | 107 | 273 | 61.9 | 93.5 | 56.1-67.7 | 88.8-98.1 | 0.41 | 9.46 | 0.35-0.48 | 4.60-19.48 | NA** | NA** |
| **Babinski asynergy sign** ** [1, 10] | 2, 2, 2 | 91 | 60 | 81.7 | 97.8 | 71.9-91.5 | 94.8-100.0 | 0.19 | 37.16 | 0.15-0.24 | 26.82-51.48 | NA** | NA** |

***** Only a subset of studies provided all items to calculate the HINTS “plus”. Adding a fourth item (Boolean “or”) to a decision rule (compared to the three-item HINTS [without hearing]) can only decrease the specificity, thus specificity values for HINTS “plus” will be equivalent or slightly lower.

† Only studies that explicitly reported on directional changes of spontaneous nystagmus in left vs. right gaze were included, whereas studies reporting on the mere “presence vs. absence” of gaze-evoked nystagmus without providing the laterality or direction of the nystagmus were discarded. This is because unilateral gaze-evoked nystagmus (or bilateral gaze-evoked nystagmus, beating in the same direction) would not meet the criteria for this clinical finding.

‡ Different ratings for ataxia grade 1 and 2 were used in these studies. While some studies adhered to the GTI definitions as proposed by Moon and colleagues [14], who defined grade 1 as "mild to moderate imbalance with walking independently" and grade 2 as "severe imbalance with standing, but cannot walk without support" [3, 6, 7, 9, 13, 14, 19], other studies followed the GTI definitions as proposed by Lee and colleagues [12], who defined grade 1 as "sway on Romberg" and grade 2 as "able to stand but no tandem gait" [1, 2, 5, 10, 12, 16].

§ Not all studies reporting on truncal instability diagnostic accuracy indicated the anatomic location of the stroke (PICA or AICA-territory stroke patients). For the calculation of the specificity and LR+ (and associated 95% confidence intervals), we used the n’s for stroke and the n’s for pAVS from the subgroup row (PICA or AICA).

**||** There were minor differences in the definition of grade 3 truncal ataxia amongst different studies. While Lee and colleagues defined grade 3 GTI as "falling at upright posture" [12], Moon and colleagues referred to grade 3 GTI as “unable to stand or sit without support” [14].

**¶** Central-type nystagmus was defined as purely vertical, purely torsional, combined vertical-torsional (including seesaw nystagmus), horizontal-vertical spontaneous nystagmus, periodic alternating nystagmus (PAN) or gaze-evoked nystagmus.

** For these combined bedside examination approaches, too few studies provided all the numbers required to calculate reliable results using the random effect model (using the DerSimonian-Laird estimator). Therefore, these results are not shown here and non-model based (preliminary) calculations for sensitivity, specificity, LR+ and LR- are shown here instead (using excel instead). Note that the results of these calculations do not have the same rigor and thus cannot be compared directly with the other diagnostic accuracy measures.

Abbreviations: AICA=anterior inferior cerebellar artery; APV=acute peripheral vestibulopathy; cAVS=central acute vestibular syndrome; HINTS=head-impulse, nystagmus, test of skew; HINTS plus=head-impulse, nystagmus, test of skew, new hearing loss; hHIT=horizontal head-impulse test; LR+=positive likelihood ratio; LR-=negative likelihood ratio; pAVS=peripheral acute vestibular syndrome; PICA=posterior inferior cerebellar artery; SN=spontaneous nystagmus.

**References**

[1] Carmona S, Martinez C, Zalazar G, Moro M, Batuecas-Caletrio A, Luis L and Gordon C. The Diagnostic Accuracy of Truncal Ataxia and HINTS as Cardinal Signs for Acute Vestibular Syndrome. Front Neurol 2016: 7:125. doi 10.3389/fneur.2016.00125

[2] Carmona S, Martinez C, Zalazar G, Koohi N and Kaski D. Acute truncal ataxia without nystagmus in patients with acute vertigo. Eur J Neurol 2023: 30:1785-90. doi 10.1111/ene.15729

[3] Casani AP, Dallan I, Cerchiai N, Lenzi R, Cosottini M and Sellari-Franceschini S. Cerebellar infarctions mimicking acute peripheral vertigo: how to avoid misdiagnosis? Otolaryngol Head Neck Surg 2013: 148:475-81. doi 10.1177/0194599812472614

[4] Chen L, Lee W, Chambers BR and Dewey HM. Diagnostic accuracy of acute vestibular syndrome at the bedside in a stroke unit. J Neurol 2011: 258:855-61. doi 10.1007/s00415-010-5853-4

[5] Chen L, Todd M, Halmagyi GM and Aw S. Head impulse gain and saccade analysis in pontine-cerebellar stroke and vestibular neuritis. Neurology 2014: 83:1513-22. doi 10.1212/WNL.0000000000000906

[6] Choi JH, Kim HW, Choi KD, Kim MJ, Choi YR, Cho HJ, Sung SM, Kim HJ, Kim JS and Jung DS. Isolated vestibular syndrome in posterior circulation stroke: Frequency and involved structures. Neurol Clin Pract 2014: 4:410-8. doi 10.1212/CPJ.0000000000000028

[7] Choi JH, Seo JD, Choi YR, Kim MJ, Kim HJ, Kim JS and Choi KD. Inferior cerebellar peduncular lesion causes a distinct vestibular syndrome. Eur J Neurol 2015: 22:1062-7. doi 10.1111/ene.12705

[8] Choi JH, Park MG, Choi SY, Park KP, Baik SK, Kim JS and Choi KD. Acute Transient Vestibular Syndrome: Prevalence of Stroke and Efficacy of Bedside Evaluation. Stroke 2017: 48:556-62. doi 10.1161/STROKEAHA.116.015507

[9] Choi JH, Oh EH, Park MG, Baik SK, Cho HJ, Choi SY, Lee TH, Kim JS and Choi KD. Early MRI-negative posterior circulation stroke presenting as acute dizziness. J Neurol 2018: 265:2993-3000. doi 10.1007/s00415-018-9097-z

[10] Kattah JC, Martinez C, Zalazar G, Batuecas A, Lemos J and Carmona S. Role of incubitus truncal ataxia, and equivalent standing grade 3 ataxia in the diagnosis of central acute vestibular syndrome. J Neurol Sci 2022: 441:120374. doi 10.1016/j.jns.2022.120374

[11] Kim JS. Pure lateral medullary infarction: clinical-radiological correlation of 130 acute, consecutive patients. Brain 2003: 126:1864-72. doi 10.1093/brain/awg169

[12] Lee H, Sohn SI, Cho YW, Lee SR, Ahn BH, Park BR and Baloh RW. Cerebellar infarction presenting isolated vertigo: frequency and vascular topographical patterns. Neurology 2006: 67:1178-83. doi 10.1212/01.wnl.0000238500.02302.b4

[13] Liu X, Li Z, Ju Y and Zhao X. Application of bedside HINTS, ABCD(2) score and truncal ataxia to differentiate cerebellar-brainstem stroke from vestibular neuritis in the emergency room. Stroke Vasc Neurol 2024. doi 10.1136/svn-2023-002779

[14] Moon IS, Kim JS, Choi KD, Kim MJ, Oh SY, Lee H, Lee HS and Park SH. Isolated nodular infarction. Stroke 2009: 40:487-91. doi 10.1161/STROKEAHA.108.527762

[15] Nam GS, Shin HJ, Kang JJ, Lee NR and Oh SY. Clinical Implication of Corrective Saccades in the Video Head Impulse Test for the Diagnosis of Posterior Inferior Cerebellar Artery Infarction. Front Neurol 2021: 12:605040. doi 10.3389/fneur.2021.605040

[16] Newman-Toker DE, Kerber KA, Hsieh YH, Pula JH, Omron R, Saber Tehrani AS, Mantokoudis G, Hanley DF, Zee DS and Kattah JC. HINTS outperforms ABCD2 to screen for stroke in acute continuous vertigo and dizziness. Acad Emerg Med 2013: 20:986-96. doi 10.1111/acem.12223

[17] Kattah JC, Talkad AV, Wang DZ, Hsieh YH and Newman-Toker DE. HINTS to diagnose stroke in the acute vestibular syndrome: three-step bedside oculomotor examination more sensitive than early MRI diffusion-weighted imaging. Stroke 2009: 40:3504-10.

[18] Ogawa K, Suzuki Y, Takahashi K, Akimoto T, Kamei S and Soma M. Clinical Study of Seven Patients with Infarction in Territories of the Anterior Inferior Cerebellar Artery. J Stroke Cerebrovasc Dis 2017: 26:574-81. doi 10.1016/j.jstrokecerebrovasdis.2016.11.118

[19] Ye BS, Kim YD, Nam HS, Lee HS, Nam CM and Heo JH. Clinical manifestations of cerebellar infarction according to specific lobular involvement. Cerebellum 2010: 9:571-9. doi 10.1007/s12311-010-0200-y
